# Supplementary material for: Early presence of Homo sapiens in Southeast Asia by 86–68 kyr at Tam Pà Ling, Northern Laos
Source: Nat Commun. 2023 Jun 13;14:3193. doi: 10.1038/s41467-023-38715-y (PMC10264382; doi:10.1038/s41467-023-38715-y)
Supplement: Supplementary file 1 — Supplementary Information [file 41467_2023_38715_MOESM1_ESM.pdf]

Supplementary Information

Early presence of *Homo sapiens* in Southeast Asia by 86-68 kyr at Tam Pà Ling, Northern Laos.

Sarah E. Freidline[1, 2], Kira Westaway[3], Renaud Joannes-Boyau[4, 5], Philippe Duringer[6], Jean-Luc Ponche[7], Mike W. Morley[8], Vito C. Hernandez[8], Meghan S. McAllister-Hayward[8], Hugh McColl[9], Clément Zanolli[10], Philipp Gunz[2], Inga Bergmann[2], Phonephanh Sichanthongtip[11], Daovee Sihanam[11], Souliphane Boualaphane[11], Thonglith Luangkhoth[11], Viengkeo Souksavatdy[11], Anthony Dosseto[12], Quentin Boesch[6], Elise Patole-Edoumba[13], Françoise Aubaile[14], Françoise Crozier[15], Eric Suzzoni[16], Sébastien Frangeul[16], Nicolas Bourgon[2,17], Alexandra Zachwieja[18] Tyler E. Dunn[19], Anne-Marie Bacon[20], Jean-Jacques Hublin[21,2], Laura Shackelford[22, 23*], Fabrice Demeter[9, 14*]

[1] Department of Anthropology, University of Central Florida, 4000 Central Florida Blvd., Howard Phillips Hall, Orlando, USA

[2] Department of Human Origins, Max Planck Institute for Evolutionary Anthropology, Deutscher Platz 6, Leipzig, Germany

[3] School of Natural Sciences, Faculty of Science and Engineering, Macquarie University, NSW 2109, Australia

[4] Geoarchaeology and Archaeometry Research group (GARG), Southern Cross University, NSW, Australia

[5] Centre for Anthropological Research, University of Johannesburg, Gauteng Province, South Africa

[6] Ecole et Observatoire des Sciences de la Terre, Institut de Physique du Globe de Strasbourg (IPGS), UMR 7516 CNRS, Université de Strasbourg, France

[7] Université de Strasbourg, Laboratoire Image, Ville Environnement, UMR 7362 UdS CNRS, France

[8] Flinders Microarchaeology Laboratory, Archaeology, College of Humanities and Social Sciences, Flinders University, Sturt Road, Bedford Park, Adelaide, Australia

[9] Lundbeck Foundation GeoGenetics Centre, Globe Institute, University of Copenhagen, Copenhagen, Denmark

[10] Univ. Bordeaux, CNRS, MCC, PACEA, UMR 5199, 33600 Pessac, France

[11] Ministry of Information, Culture and Tourism, Laos PDR

[12] Wollongong Isotope Geochronology Laboratory, School of Earth, Atmospheric & Life Sciences, University of Wollongong, NSW, Australia

[13] Muséum d'Histoire naturelle de La Rochelle, France

[14] Eco-anthropologie (EA), Muséum national d'Histoire naturelle, CNRS, Université de Paris, Musée de l'Homme 17 place du Trocadéro 75016 Paris, France

[15] IRD, DIADE, Montpellier, France

[16] Spitteurs Pan, technical cave supervision and exploration, La Chapelle en Vercors, France

[17] Applied and Analytical Palaeontology, Institute of Geosciences, Johannes Gutenberg University, 55128 Mainz, Germany

[18] Department of Biomedical Sciences, University of Minnesota Medical School, Duluth, MN, USA

[19] Anatomical Sciences Education Center, Oregon Health & Sciences University, Portland, OR, USA

[20] Université Paris Cité, BABEL CNRS UMR 8045 Paris, France

[21] Chaire de Paléoanthropologie, CIRB (UMR 7241 – U1050), Collège de France. 11, Place Marcelin-Berthelot, 75231 Paris Cedex 05, France

[22] Department of Anthropology, University of Illinois at Urbana-Champaign, Urbana, IL, USA

[23] Carle Illinois College of Medicine, University of Illinois at Urbana-Champaign, Urbana, IL, USA

This PDF file includes:

Supplementary Discussion

Supplementary Figures 1 to 27

Supplementary Table 1

Supplementary References

Supplementary discussion

Location

Tam Pà Ling (TPL) is located in northeast Laos, about 260 km NNE from Vientiane, in the Huà Pan Province (Supplementary Fig. 1). The cave is situated in a limestone karstic tower known as Pà Hang, located on the south-eastern side of P'ou Loi Mountain. The latter belongs to the septentrional Annamitic Chain, oriented broadly NNW-SSE. The nearest major city is Vieng Thong (Supplementary Fig. 1), also known as Ban That Hium on Google Earth mapping. From there, the site can be reached traveling north by road for ~20 km along the Nam Kam River to the small Hmong village of Long Nguapha. Surrounding Pà Hang, there are multiple cavities explored by us in the past and located in a radius of 1 km from the center of the village.

Geology

The geology of TPL consists of Palaeozoic granite and diorite[1] covered with a widespread folded poorly metamorphic sedimentary formation characterized by gray to yellow pelites, siltites and arkosic sandstones attributed to the Silurian, and perhaps partly to the Devonian[1]. This poorly metamorphic unit is capped by a thick limestone unit, which is badly dated at present, but which has been attributed by Saurin[1] to an

interval between the Late Carboniferous (Moscovian) and the Permian. The limestone unit consists of a pluri-decametric massive sparitic dark-gray carbonate without marl intercalation. Coral fossils are quite common according to place. On the way to the fossiliferous sites starting from Vieng Thong, the road successively incises through the three main formations of the region, namely the granite massif, the weakly metamorphic folded series, and the carbonate unit. In the studied region, the landscape consists of tower karsts formed through the dissolution of Moscovian limestone beds, containing a dense network of caves and galleries. Most tower karsts in the region display widespread karstic networks and at present at least half a dozen sites rich in fossils have been discovered and are in the process of being analyzed.

The TPL cavity (Cave of the Monkeys -20° 12′ 31.4″ N, 103 ° 24′ 35.2″ E) is located on the top of the Pa Hang Mountain 1,170 m above sea level. It has one main chamber (40 m width × 30 m length × 10-18 m height) with a south-facing large entrance (Supplementary Fig. 2). From the main chamber, close to the roof, exists a second network ~5 to 10 m wide, 2 to 4 m high and 100 m long. At the end of the gallery, a very narrow gallery ~20 m long crosses the mountain to another outside exit (Supplementary Fig. 2).

In the main entrance and down the main talus slope, the presence of large limestone blocks and slabs testify to collapse of the roof of the cave through slab failure, allowing the formation of the current large opening of the cave. The collapse of a part of the roof at the entrance is argued by a cutting of the roof according to the directions of diaclases as well as by the presence of stalactites much smaller (younger) than inside the cave (Supplementary Fig. 3). We observe layers in the stratigraphy that clearly lap up against and drape over the limestone blocks in the lower part of the sequence, consistent with the collapse of the cave preceding the deposition of the fine-grained sandy clays and clay silts. In this sense, the original cave floor strewn with limestone slabs formed the topographic template that governed subsequent sedimentation. Further, we do not record signs of deformation of the fine-grained units beneath or adjacent to the large limestone slabs, which would be expected if they had fallen on to this rather plastic sediment.

In 2008, three test pits were opened on the cave floor to examine stratigraphy and sedimentology: Trenches 1 and 2 on the west side and Trench 3 on the east side. Trench 3, which can be reached by a steep slope ~60 m in length, is where all the fossils so far reported from TPL were found. In 2019, Trench 3 was extended east towards the wall of the cave, and by 2020, the excavators reached the limestone base of the TPL cavity, exposing a 7 m deep profile from which the stratigraphy and sedimentology is examined.

The TPL sediment consists primarily of brown silty clays that alternate with coarser, sandier layers. The sandy layers are themselves intermixed with clay and exhibit a very slight color change (Stratigraphic section Fig. 3). The main cavity of the cave does not have any other obvious connections to the wider karst system which leads us to believe that the primary or only source of sediment can be via the current main entrance. During heavy rainfall we can clearly see water running down the slope, and occasionally small ephemeral pools of water temporarily form at the base of the cave. It is evident that the sediment originated near the cave entrance and was introduced to the cave interior via relatively slow, low-energy slopewash transport (Supplementary Fig. 4), although changes in sediment characteristics indicate that higher-energy processes did sometimes occur.

Clay-rich layers contain small limestone clasts and iron-oxide pisolites. The former is derived from the host limestone bedrock (the autogenic component), whereas the quartz, clays, and secondary iron oxides are primarily derived from the platform in front of the cave entrance where these elements are present in abundance. The clay composition (vermiculite and kaolinite associated with iron and aluminum oxides) indicates hydrolysing conditions that are characteristic of "ferralsols-nitisols," which are strongly weathered soil types that suggest that they were deposited in a humid, subtropical climate.

A first profile was documented during initial excavations of Trench 3 (right part of Stratigraphic section Fig. 3). Subsequent excavations of Trench 3 near the East wall of the cave led to the documentation of a second profile, which is ~5 m from the first (left part of Stratigraphic section Fig. 3). By correlating the two profiles we find that the stratigraphy and sedimentation are consistent between the two trenches

and the individual strata and laminae are contiguous and can be followed laterally across the excavated area of the chamber; alternations of sandy and silty clay layers are punctuated by ~12 calcareous layers—similar to moon milk—which are sometimes cemented; and that there is no evidence in the excavated strata of bioturbations, post depositional modifications, soft deformations, reworking or mixing. Ongoing microstratigraphic analyses by MWM and VPCH is likely to support many of these observations.

The ongoing microstratigraphic analyses by VPCH and MWM have identified some combustion residues in the sediments sampled from the main excavation areas[2] that may suggest human activity in close vicinity of the cave or even from areas of the cave now covered in limestone slabs. Small traces of calcareous ash which are not likely to survive longer distance transport (e.g., downslope from the entrance area) as well as intact charcoal fragments that retain morphologies consistent with very short distance travel have been identified in thin section using optical microscopy and further work will quantify the amount and context of this material. However, despite ongoing microstratigraphic analyses, and the presence of the *H. sapiens* skeletal material found in the sediments, Tam Pà Ling was not likely an occupation site. No artifacts have been found in the cave and at this stage we must presume that while they were clearly out in the landscape, the cave was either not habitable or accessible at the time that these early humans were present in this area.

Magnetic Susceptibility

To assess inputs of pedogenic material from the cave mouth area, we have used magnetic susceptibility (MS) as a proxy for intensity of pedogenesis and the reworking of this material into the cave. Previously, MS has been used as a palaeoclimatic proxy in archaeological cave settings (e.g., refs.[3,4]), but these studies serve to highlight the issues with disentangling elevated MS values being related either to pedogenesis or burning occurring at the site. Warmer and wetter climatic conditions can certainly accelerate pedogenesis, forming magnetic minerals such as maghemite and magnetite and resulting in elevated MS values.

Conversely, when cooler and drier conditions prevail, a reduction in pedogenesis occurs and the MS signature is correspondingly lower[5,6]. As the East Asian Monsoon (EAM) controls much of Southeast Asian rainfall, MS was performed on bulk sediment samples from TPL to determine if—and to what extent—MS serves as a proxy for wetter or drier conditions and the flux of soil into the cave interior.

A total of 39 bulk sediment samples (18 from sediment Profile 1 and 21 from Profile 2, respectively left and right on the Stratigraphic section Fig. 3) were analyzed for MS. Samples were dried at 30°C for 72 hours. Once dry, samples were pestle and mortared (with rock fragments and/or artefacts, i.e.,shell and bone fragments, removed prior) and sieved via a 2 mm sieve. All equipment used on the samples were cleaned between each sample with Triple 7 EnviroLab LabPower, 3 rinses in water, ethanol, DI (deionized) water and acetone. Samples weighing between 29-31 grams were run on a Bartington *MS2* Magnetic Susceptibility System as detailed in Dearing[7], using standard international (SI) units. Low frequency (LF) results are displayed in Supplementary Fig. 5 as it has the highest sensitivity to magnetic changes[4].

MS values support observations that periods of increased precipitation (resulting in increased pedogenesis) correlate with greater soil delivery to the interior of the cave from the cave mouth area during periods of greater rainfall and slopewash activity. This is supported by the rise in MS values between 5.32-3.72 m (P2), with sediment at these depths comprising predominantly silty clays (Stratigraphic section Fig. 3). The same pattern is observed between 3.4 – 1.8 m (P1) and 3.52 – 1.92 m (P2), where higher MS values occur when sediment is predominantly composed of silty clays. We note that higher MS values are less pronounced in P2 than P1, likely due to the fact these sediment columns are laterally 5 m apart (Stratigraphic section Fig. 3) and therefore other spatial aspects are controlling sediment delivery.

The MS data suggest a decline in overall moisture availability during much of MIS 4, potentially linked to higher seasonal variability related to a weakening of the EAM. A similar decline in rainfall in MIS 4 is also observed by Bacon et al.[8] at Duoi U'Oi, northern Vietnam. Wetter conditions return towards the MIS 4–3 transition related to a strengthening of the EAM, which aligns well with similar interpretations by

Milano et al.[9]. A steady rise in MS values during MIS 3 (peaking around 2.8 m, ~46 ka), declining towards the end of MIS 3 and into MIS 2 also corresponds with the general drying trend observed by Milano et al.[9] at Tam Pà Ling and Yamaoh et al.[10] at Lake Pa Kho, northeastern Thailand. This shift towards drier conditions is recorded slightly later at Tham Lod (noting this record only extends to 34.1 ka), with Marwick and Gagan[11] determining a wet and unstable climate persisted during 33 – 20 ka, beginning to shift towards drier conditions between 20 – 11.5 ka. The notable decline in moisture availability during MIS 2 recorded by both Milano et al and Marwick & Gagan is also observed in this TPL MS record. Towards the end of MIS 2 and into the Holocene, MS values begin a staggered but steady rise, indicating a return of wetter conditions, likely due to strengthening of the EAM.

Bulk organic and carbonate content (Loss on ignition)

To further interrogate the formation of the TPL stratigraphy, the loss-on-ignition (LOI) technique was applied to TPL bulk sediment samples to provide a broad estimate of the amounts of organic matter (OM) and carbonate content (CC) in the sediment[12-14]. When combined with other sedimentological parameters, such as magnetic susceptibility (MS), LOI data can assist in determining the source and character of sediments deposited in caves.

LOI was undertaken on the same 39 bulk samples analysed for MS following standard protocols[14] using a time-programmed Across International CF1200 series muffle furnace with a 36-L chamber capacity. Results were calculated using the below equations from the same protocol[14] and presented in Supplementary Tables 1 and 2.

For OM: $LOI_{550} = ((DW_{105} – DW_{550})/DW_{105}) *100$

For CC: $LOI_{950} = ((DW_{550} – DW_{950})/DW_{105}) *100$

The difference between amounts of OM and CC at similar depths in each profile are due to their positions in the cave (Stratigraphic section Fig. 3). The higher CC in Profile 2 is attributed to its location near the cave wall, with more calcium carbonate being transferred into the sediment in this area of the site. Conversely, the higher OM in Profile 1 is most likely related to its location towards the base of the slope beneath the cave entrance, which regularly receives allogenic clay-rich colluvial slopewash sediments (Supplementary Fig. 4).

Values of OM and CC (Supplementary Fig. 6) recorded between ~6.92 – 5.52 m suggest relatively dry colluvial input, probably originating from in and around the cave entrance (Supplementary Fig. 4). This is, punctuated by cave roof slab failure during MIS 4, and the associated deposition of calcareous sediments, probably as crushed and comminuted rock powder and small limestone clasts (Stratigraphic section Fig. 3). The OM and CC values at ~5.32 – 3.72 m (Supplementary Fig. 6) are indicative of wetter, low-energy sedimentation at around ~62 – 50 ka (MIS 4 – 3). The higher OM content in sediment deposited between 3.5 – 1.9 m (~51–41 ka and 50–42 ka) suggests a shift towards dominantly wet colluviation from the entrance of the cave during MIS 3. Notably, the greater amount of CC than OM at ~2.32 m in Profile 2, and increased amount of CC in Profile 1 at ~2.2 m, may be due to a collapse of the cave roof occurring between ~50 – 42 ka and 36 – 30 ka (Supplementary Fig. 4). Major collapse events are evidenced by the large limestone boulders recorded in the site (Stratigraphic section Fig. 3). The highest OM and CC values occur ~1.67 m and ~1.4 m in Profile 1 ~25 – 21 ka and 16 – 12 ka and correlate with the alternating sandy and silty clay infilling showing slight color changes (Stratigraphic section Fig. 3). When interpreted together with increasing humidity shown by MS (associated with the strengthening of the EAM during MIS 2–1) (Supplementary Fig. 6) this likely represents periods of increased pedogenesis.

The elevated amounts of CC we record in the sediment (Supplementary Fig. 6) coincide with drier conditions as observed from the MS data. This is characterized in the stratigraphy by increased frequency of limestone clasts which may relate to periods of increased slab failure (autogenic limestone generation) (Stratigraphic section Fig. 3). These are often immediately followed or accompanied by an increase in the

amounts of OM such as ~6.52, 5.92, and 5.52 m in Profile 2, and ~3.4 and 2.2 m in Profile 1 suggesting that these slab failures in the sequence relate to episodes of cave opening which then respectively allows greater deposition of organic-rich clays further into the cave. The observations on these aspects of the sedimentological record of TPL support the idea that it was a collapse of the cave ceiling that formed its large cave opening. Although these also suggest that it took several of these events to gradually open the cave to its current size beginning from at least ~86 ± 8 kyr.

Fauna from Tam Pà Ling

Tam Pà Ling yielded abundant microvertebrates (rodents, insectivores, reptiles, and amphibians)[15], but few remains of large mammals. Since 2010, only one unidentified fragmentary long bone and twenty-eight teeth belonging to Caprinae, Cervidae, *Hystrix* sp., *Muntiacus* sp., *Macaca* sp., *Rhinoceros* cf. *unicornis*, *Dicerorhinus sumatrensis*, have been recovered in the ~7 meters high stratigraphy. Among these twenty-eight teeth, only a lower third molar of *Capricornis* sp. was still fixed in a fragmentary jawbone, the others being isolated teeth.

Regarding the two teeth used for dating, TPL 73 is a germ of a lower molar (M_1 or M_2) with an incomplete crown, whereas TPL 74 is a lower molar (M_1 or M_2) displaying incomplete roots partly gnawed by rodents. Both molars show the typical morphological pattern of Caprinae, with smooth enamel (not ridulated as in cervids), and stylids more prominent than main cusps (metaconid and entoconid). On TPL 74, the ectostylid is absent.

There is no evidence that these Caprinae could have been prey hunted by the *Homo sapiens* individuals recovered at the same level in the TPL stratigraphy. There are no archaeological artifacts, no animal bones with cut marks, no traces of human activities, and the analysis of deposits suggests that the cave was not an occupation site. The presence of teeth of large mammals results from the same depositional process as that of human remains in the cave. Moreover, the roots of most isolated teeth of large mammals show gnawing traces of rodents, most likely porcupines, which indicates that they were

the last accumulator agents. Such traces of activities of porcupines are a common phenomenon[16], as observed in the mammalian assemblages in the same area, Nam Lot (86-72 ka) and Tam Hang South (94-60 Ka)[17], from breccias deposits of the Pà Hang mountain[18]. The animal teeth, being gnawed, and the human remains, showing no trace of porcupine activity, document different taphonomic histories, which remain difficult to explain and could lead to the assumption that the human remains have been buried outside of the cave[19-21] and later washed in, which would only be putative at this stage of the analysis. However, their presence along the 7meter high section of Tam Pà Ling results most likely from the same depositional process with clay-rich slopewash deposits[15].

Supplementary Figures 1 to 28

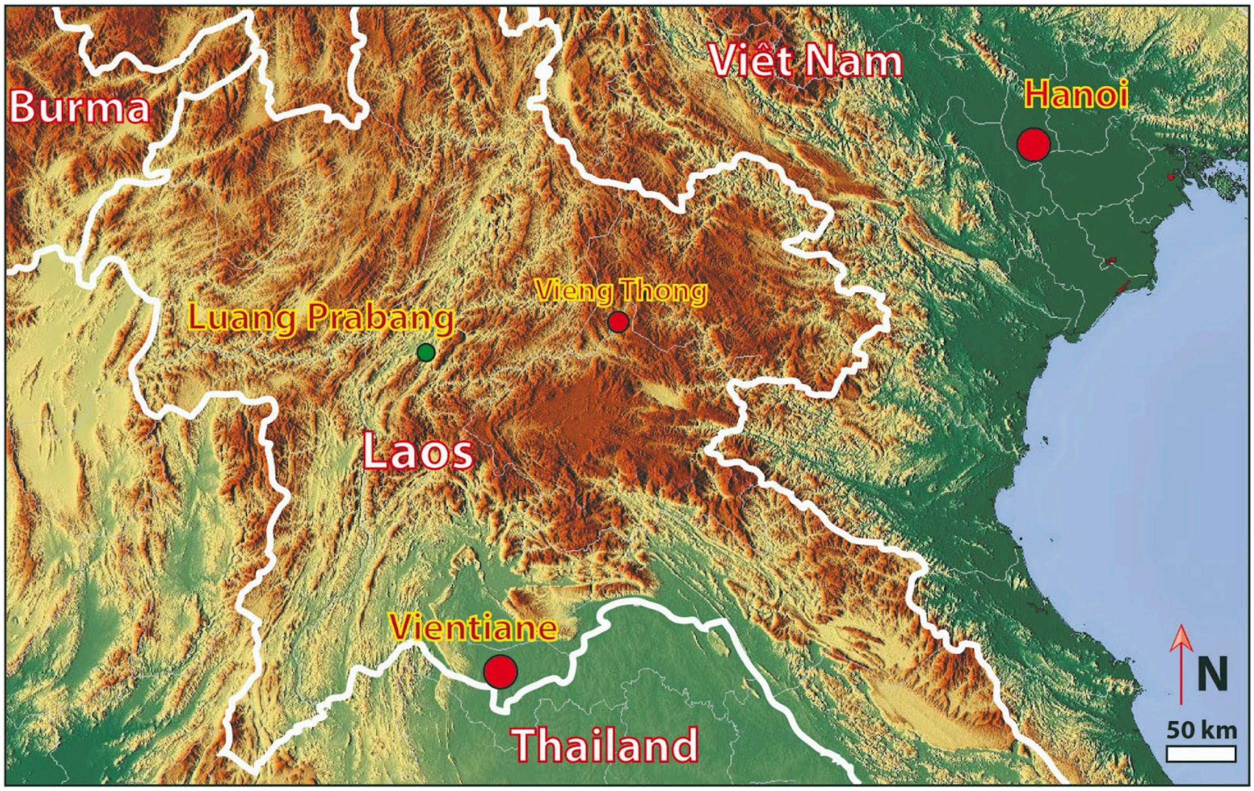

Supplementary Figure 1. Location of the site of Tam Pà Ling near Vieng Thong village (http://www.maps-for-free.com/).

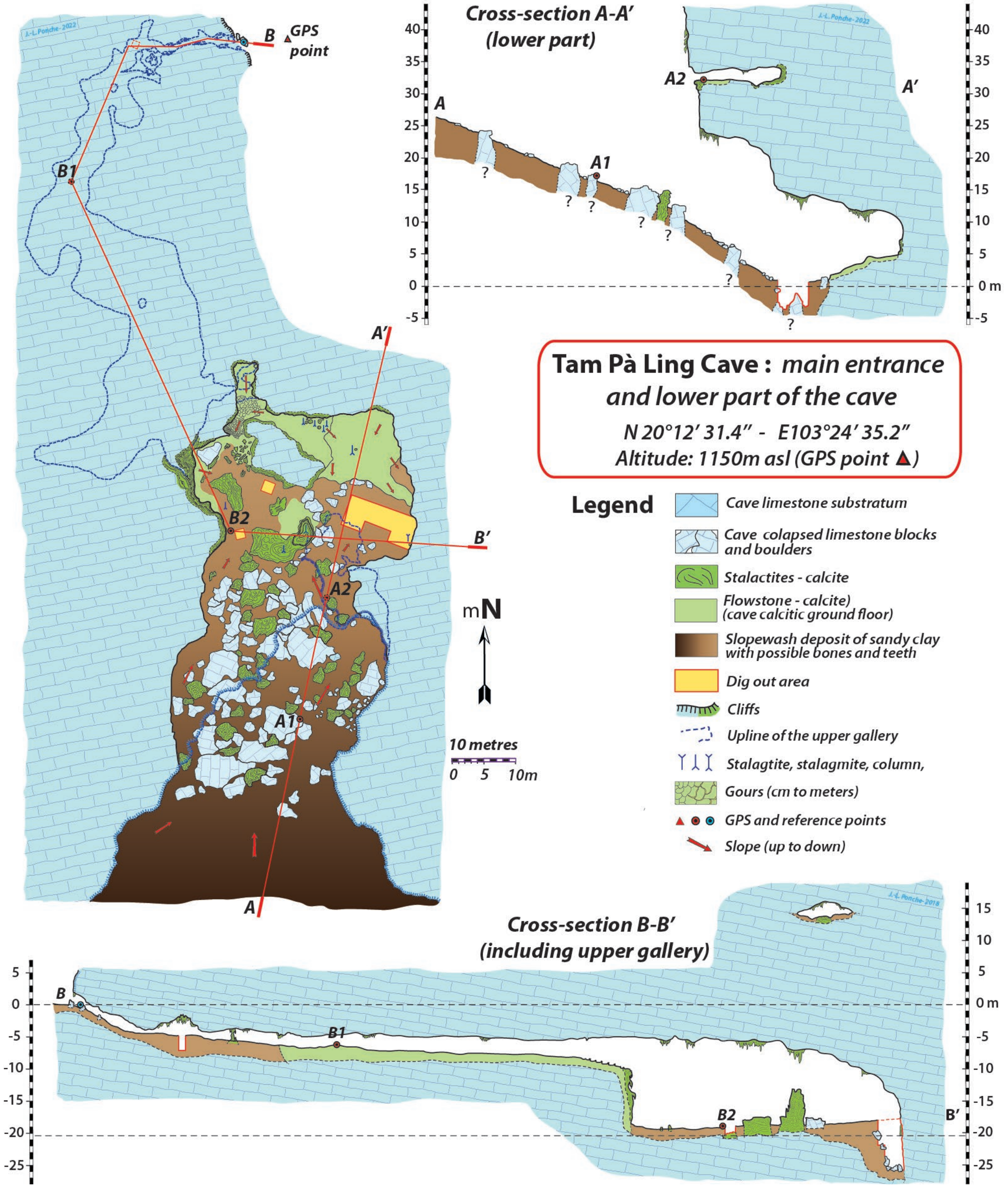

Supplementary Figure 2. Map of the cave of Tam Pà Ling.

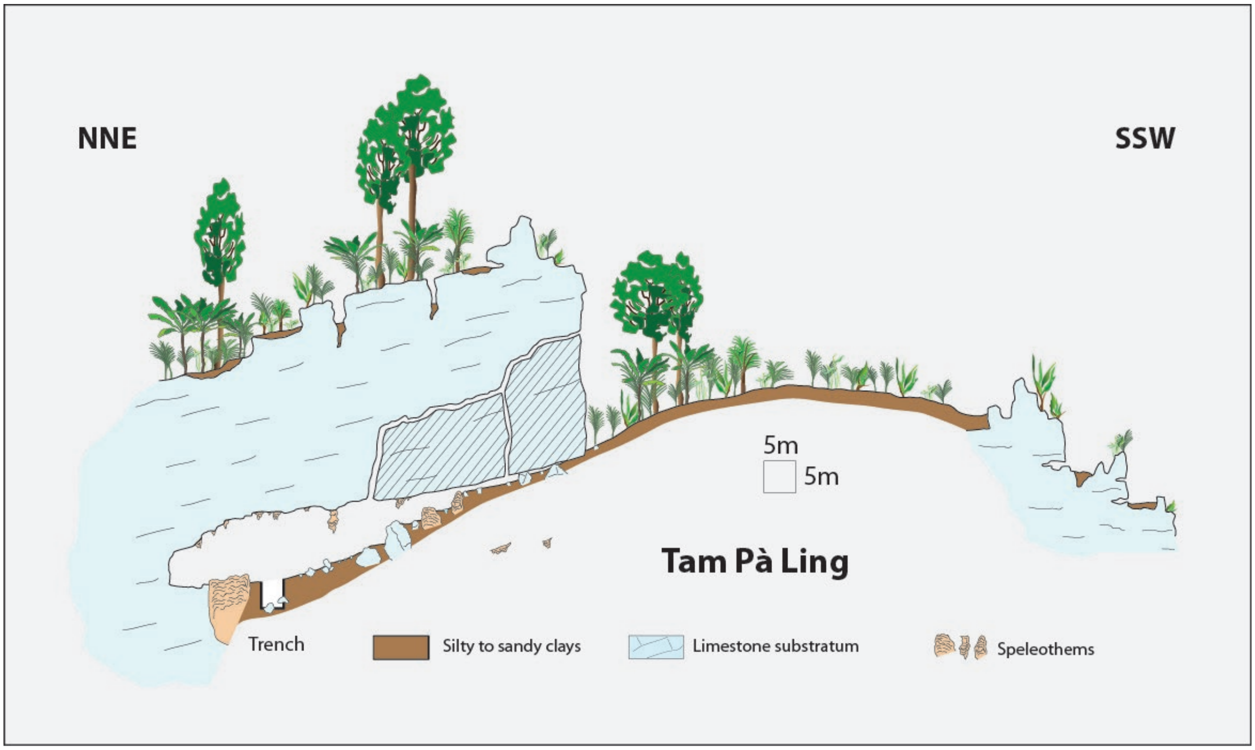

Supplementary Figure 3. Possible model of the collapse of the southern part of the main entrance of the cave which allowed the opening of the site.

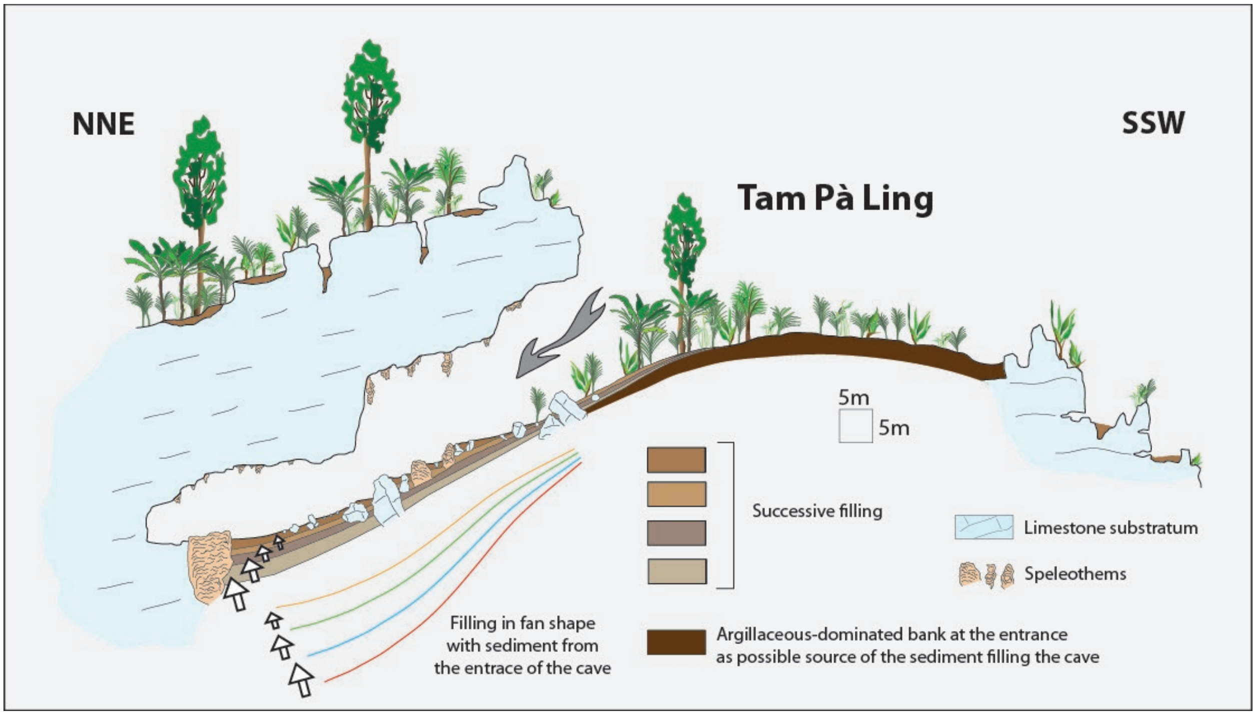

Supplementary Figure 4. Possible mechanism of filling the cave from the erosion of sediment accumulated at the entrance of the cave.

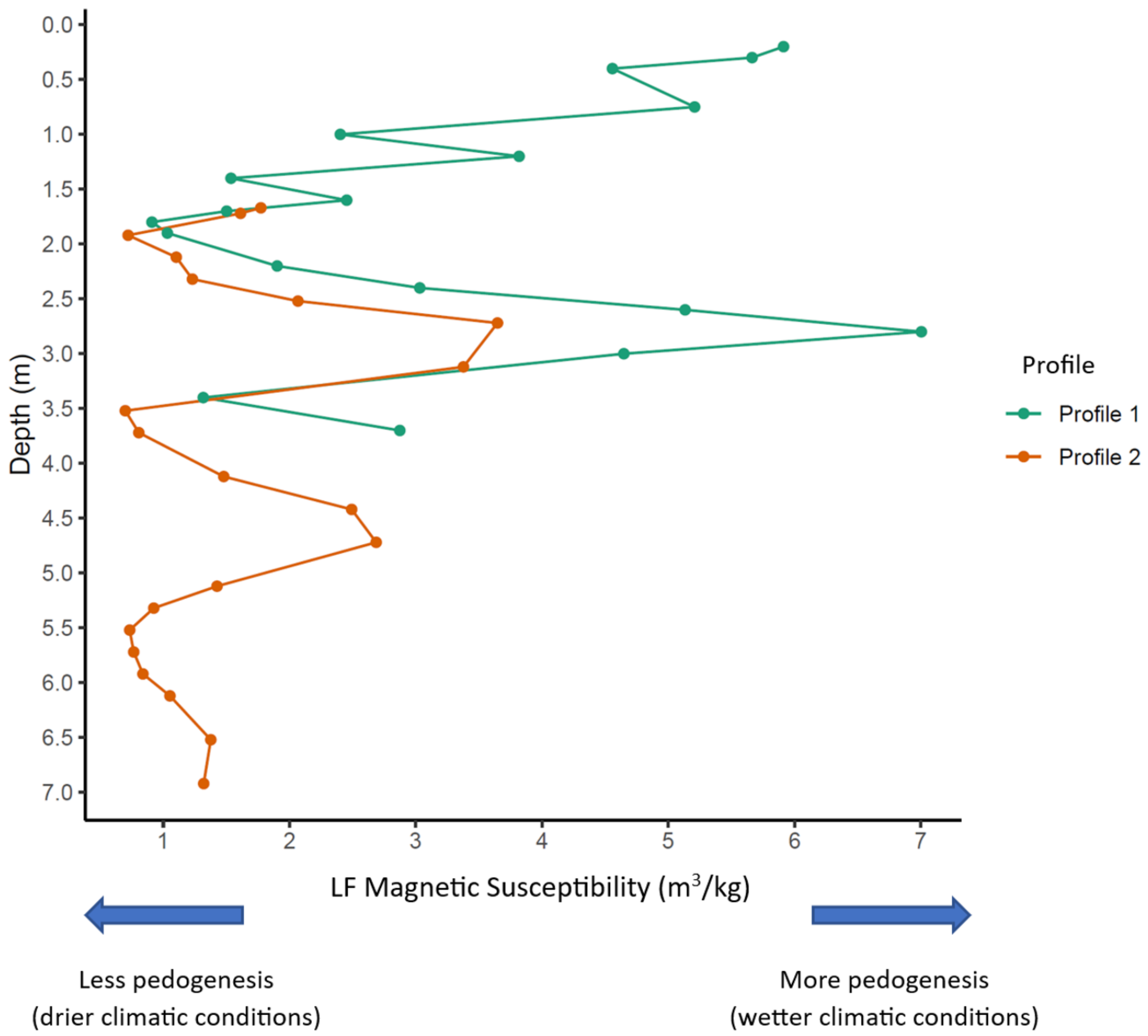

Supplementary Figure 5. Low frequency (LF) magnetic susceptibility results for profile 1 (P1; 0.2-3.7 m below ground level [bgl.]) and profile 2 (P2) (1.67-6.92 m bgl). Samples from both P1 and P2 are taken to investigate the potential correlation of sediment characteristics, given their similar deposition history (Stratigraphic section Fig. 3).

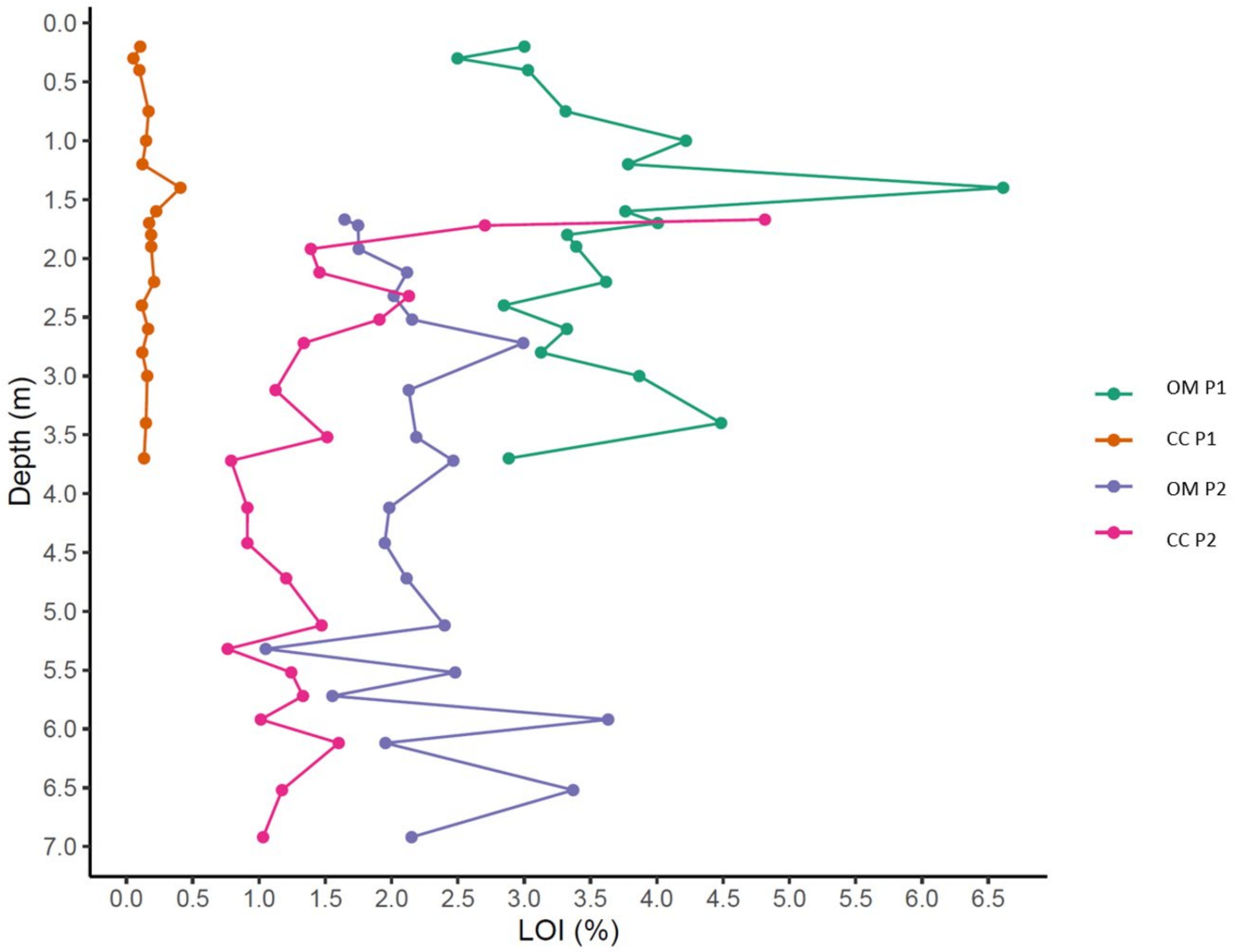

Supplementary Figure 6. Results of loss-on-ignition (LOI) from Trench 3, showing % organic matter (OM) and % carbonate content (CC) % in sediments from Profile 1 (P1, 0.2-3.7 m) below ground level [bgl] and Profile 2 (P2, 1.67-6.92 m below ground level [bgl]) (Fig. 4).

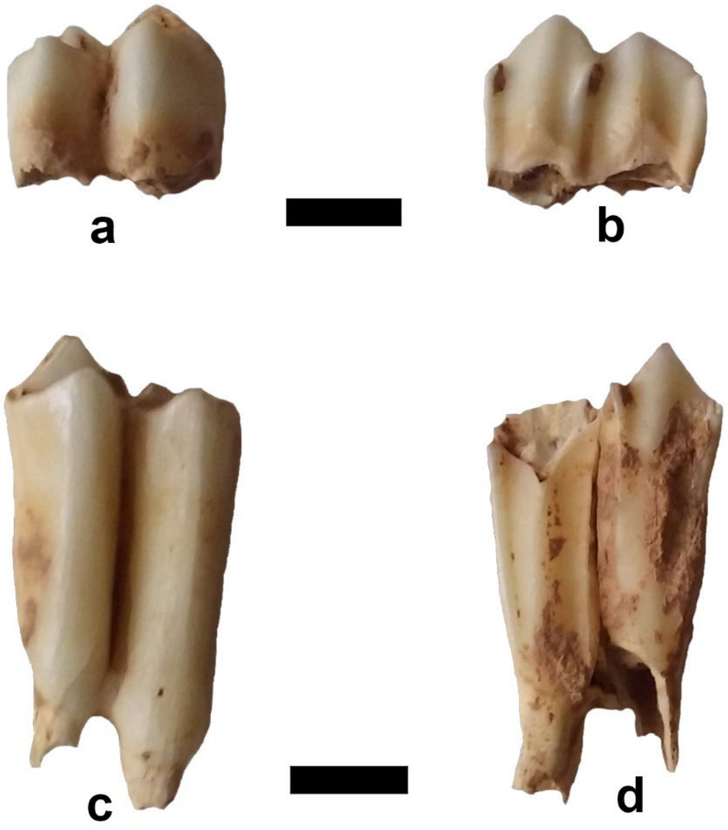

Supplementary Figure 7. Teeth used from U-series/ESR dating. ESR TPL-01 (TPL-73), (a) buccal view and (b) lingual view of a lower molar (m1/m2) of Caprinae recovered in the Tam Pà Ling stratigraphic sequence at a depth of 6.40 m. ESR TPL-02 (TPL-74), (c) buccal view and (d) lingual view of a lower molar (m1/m2) of Caprinae recovered at a depth of 6.67 m.

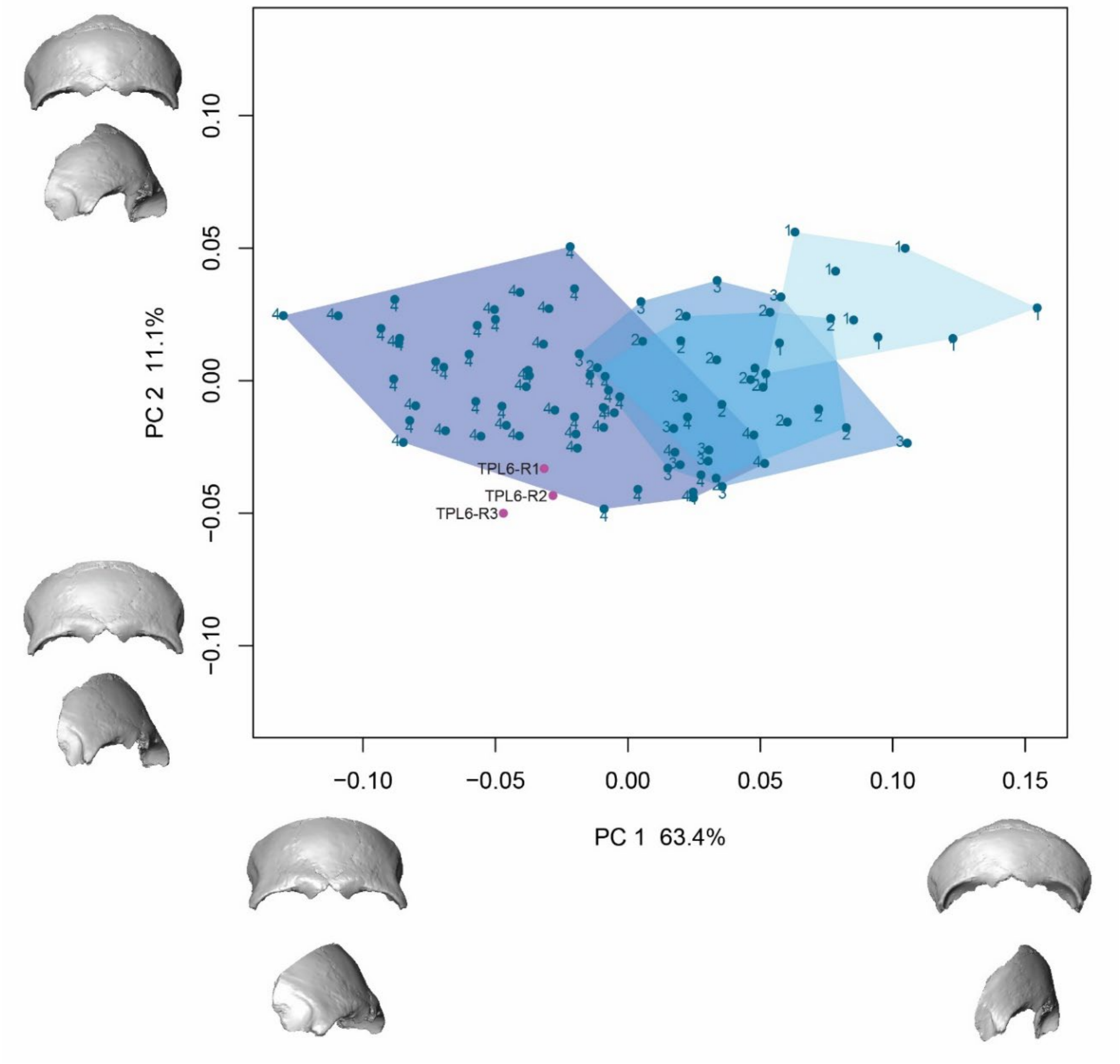

Supplementary Figure 8. Principal component analysis of frontal shape in *H. sapiens* ontogenetic series. TPL 6 (3 reconstructions TPL 6-R1, R2, R3) were projected into the plot; Age Group (AG) 1 labeled 1; AG 2 = 2; AG3 = 3; AG 4 = 4. Shape changes were visualized along PC 1 and PC 2 by warping the sample mean shape along the positive and negative ends of PC 1 and PC 2, plus/minus two standard deviations from the sample mean. Subadults associated with AG 1, plotting along the positive end of PC 1, have a more vertical frontal bone, rounded supraorbital margins, and an undeveloped brow ridge. Whereas adults, plotting at the negative end of PC 1, have a receding frontal, squarer orbits, and a more projecting brow ridge. TPL 6 resembles the adult shape. Source data are provided as a Source Data file.

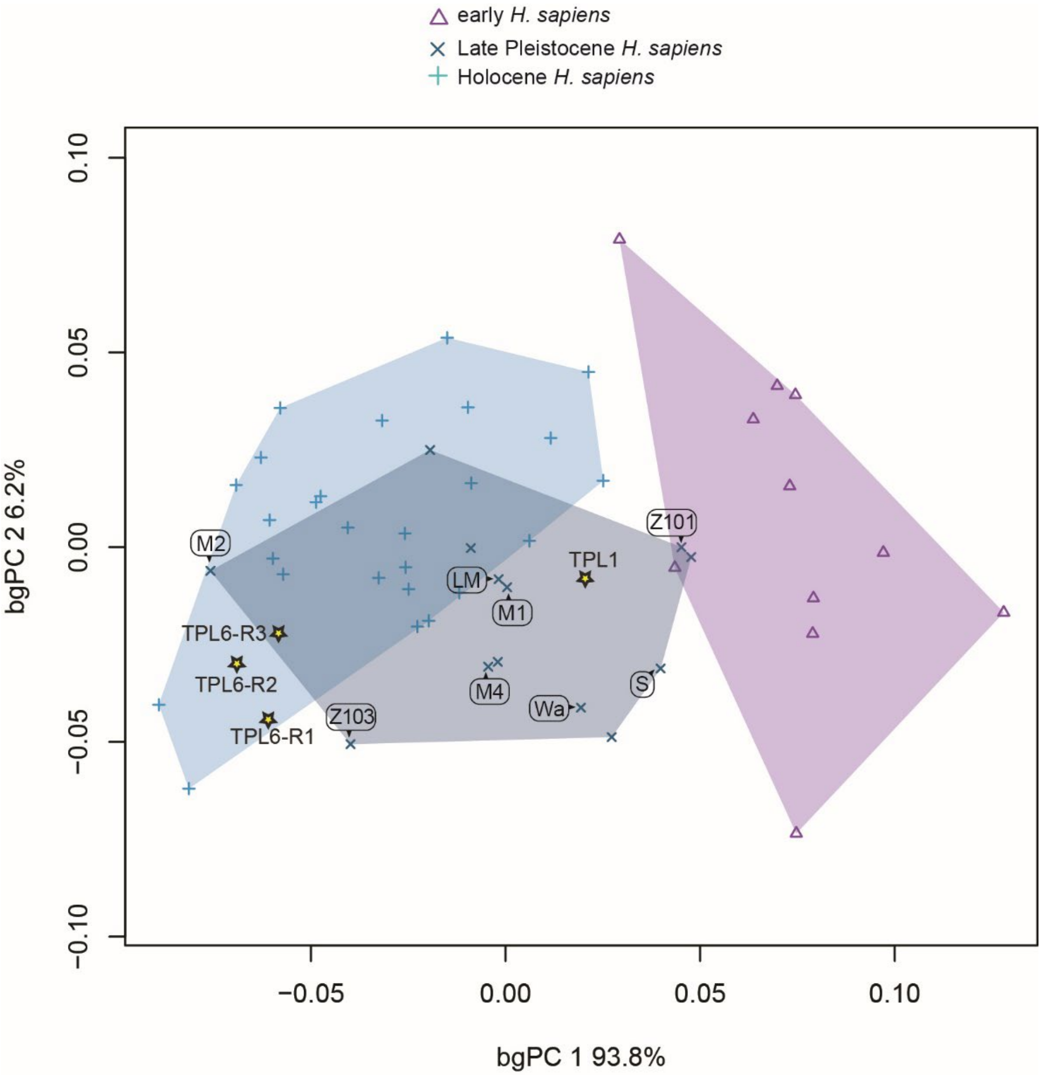

Supplementary Figure 9. Between group PCA (bgPCA) of frontal shape in *H. sapiens*. TPL 1 and TPL 6 (3 reconstructions TPL6-R1, R2, R3) were projected into the plot. Labels: Lake Mungo (LM); Minatogawa 1 (M1), 2 (M2), and 4 (M4); S (Salkhit); Wadjak 2 (Wa); and Zhoukoudian Upper Cave 101 (Z101) and 103 (Z103). Source data are provided as a Source Data file.

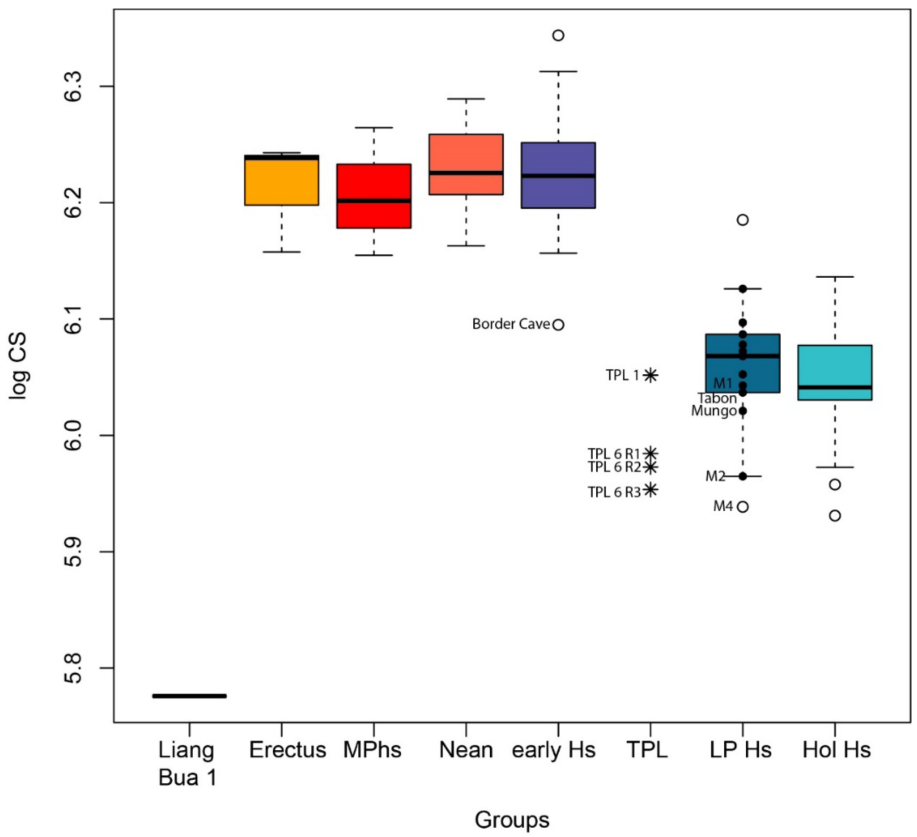

Supplementary Figure 10. TPL 1 and 6 frontal centroid sizes. Box plots depicting log centroid size for each group (*H. floresiensis* [Liang Bua 1] n = 1; *H. erectus* [Erectus] n = 3; Middle Pleistocene hominin [MPhs] n = 3; Neanderthal [Nean] n = 16; early *H. sapiens* [early Hs] n = 11; Tam Pà Ling hominins [TPL] n = 4; Late Pleistocene *H. sapiens* [LP Hs] n = 13; Holocene *H. sapiens* [Hol Hs] n = 26). Horizontal lines represent the median of each group. Boxes show the interquartile range (IQR, 25th to 75th percentile). Whiskers extend to 1.5 times IQR. Outliers are represented by circles. There is a clear difference in brow ridge size between archaic humans, including early *H. sapiens*, and later humans, and the TPL fossils fall within the range of the latter. TPL 1 is larger than TPL 6 (3 reconstructions TPL6-R1, R2, R3), falls within the interquartile range of Late Pleistocene and Holocene humans, and is comparable in size to Minatogawa 1 (M1). The TPL 6 frontal, on the other hand, is small, and comparable in size to Late Pleistocene Minatogawa 4 (M4) and A (M2) and two Holocene individuals. Source data are provided as a Source Data file.

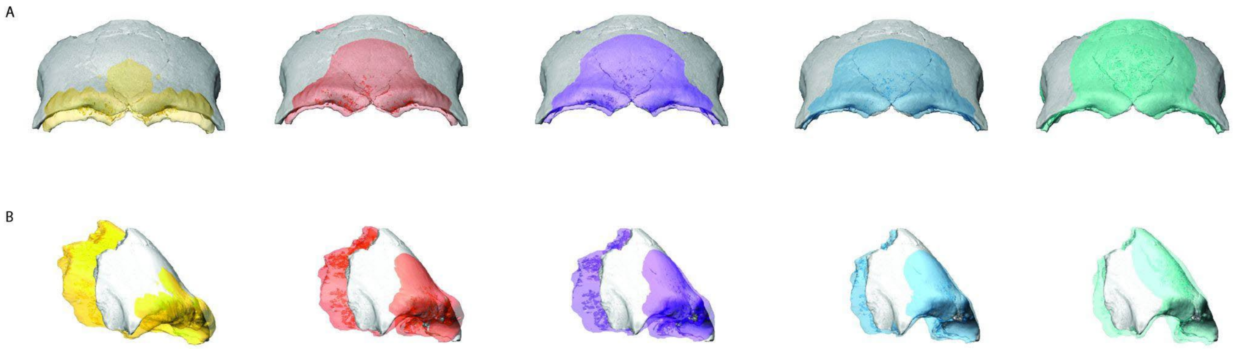

Supplementary Figure 11. TPL 6 frontal superimposed onto group mean shapes. TPL 6 (gray) superimposed on group mean shapes, anterior (A) and lateral (B) views (yellow: *H. erectus*; red: Neanderthal; purple: early *H. sapiens*; blue: Late Pleistocene Asian *H. sapiens*; green: Holocene *H. sapiens*). TPL 6 has a gracile brow ridge. Compared to all groups, its frontal bone is wider with a flatter frontal squama, and it has a more projecting lateral brow ridge. It is most similar to Late Pleistocene Asian and Holocene *H. sapiens*.

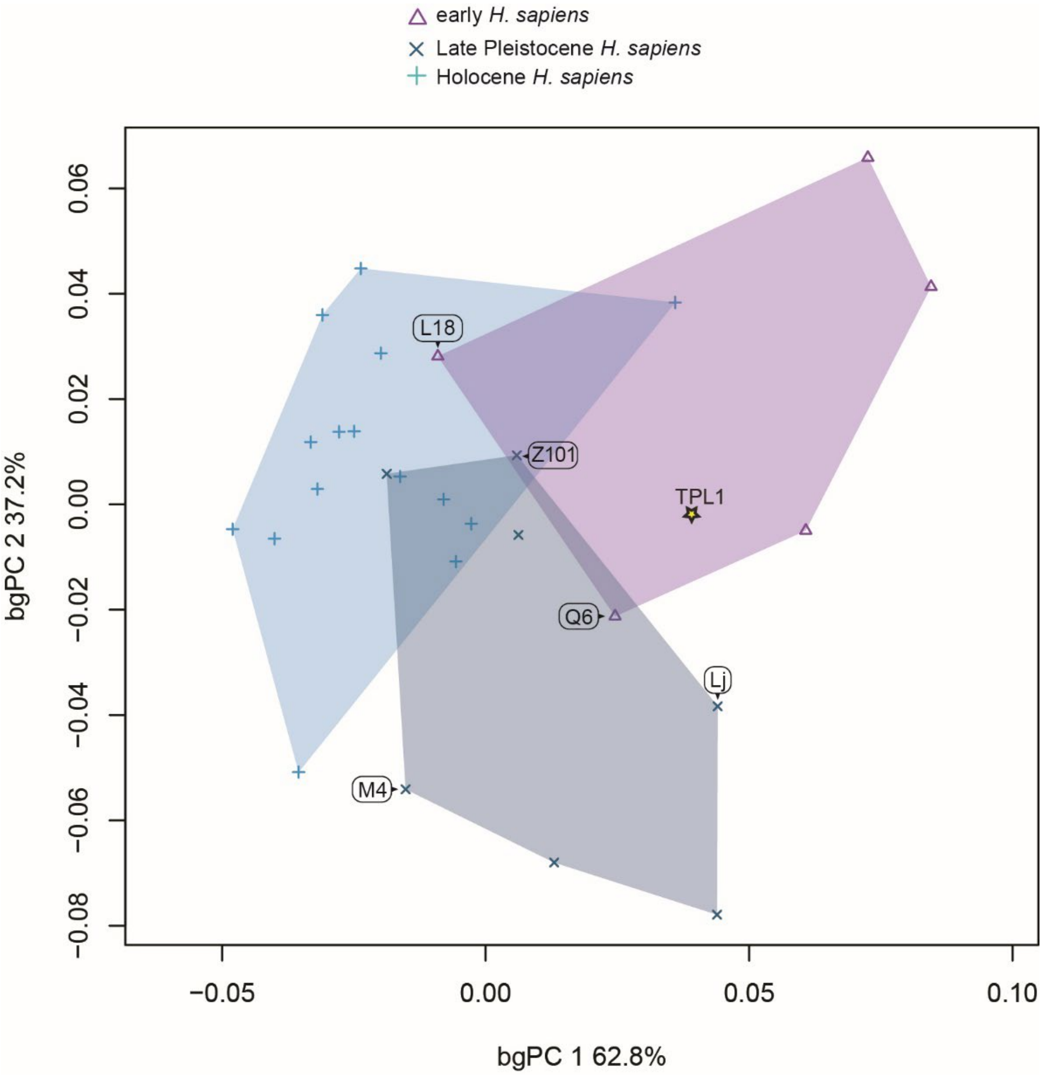

Supplementary Figure 12. Between group PCA (bgPCA) of maxillary shape in *H. sapiens*. TPL 1 was projected into the plot. Labels: Laetoli Hominin 18 (L18); Liujiang (Lj); Minatogawa 4 (M4); Qafzeh 6 (Q6); and Zhoukoudian Upper Cave 101 (Z101). Source data are provided as a Source Data file.

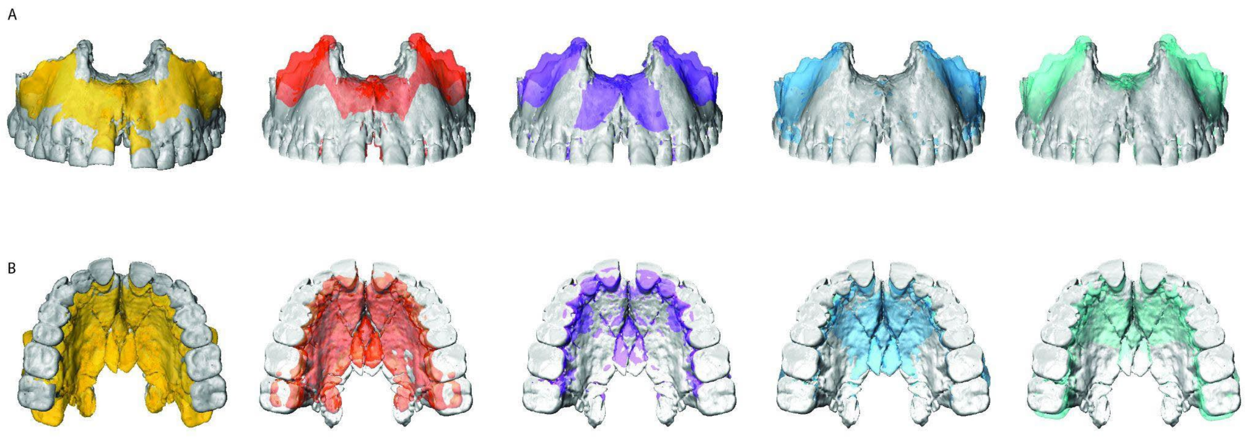

Supplementary Figure 13. TPL 1 maxilla superimposed onto group mean shapes. TPL 1 (gray) superimposed on group mean shapes, anterior (A) and lateral (B) views (yellow: *H. erectus*; red: Neanderthal; purple: early *H. sapiens*; blue: Late Pleistocene Asian *H. sapiens*; green: Holocene *H. sapiens*).

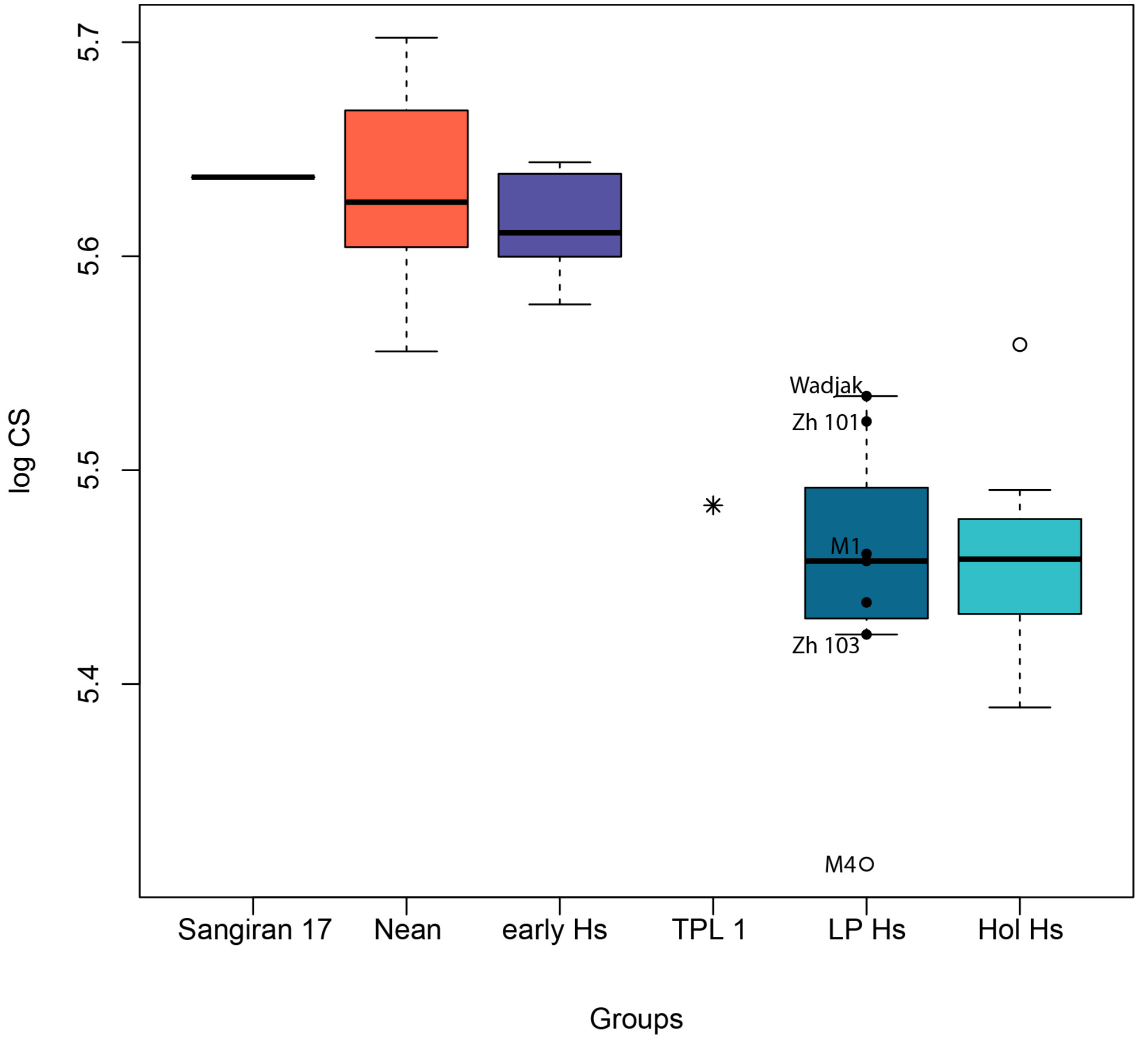

Supplementary Figure 14. TPL 1 maxilla centroid sizes. Box plots depicting log centroid size for each group (*H. erectus* [Sangiran 17] n = 1; Neanderthal [Nean] n = 9; early *H. sapiens* [early Hs] n = 5; Tam Pà Ling hominin [TPL 1] n = 1; Late Pleistocene *H. sapiens* [LP Hs] n = 7; Holocene *H. sapiens* [Hol Hs] n = 15). Horizontal lines represent the median of each group. Boxes show the interquartile range (IQR, 25th to 75th percentile). Whiskers extend to 1.5 times IQR. Outliers are represented by circles; Zhoukoudian Upper Cave 101 (Zh 101) and 103 (Zh 103), Minatogawa 1 (M1) and 4 (M4). Source data are provided as a Source Data file.

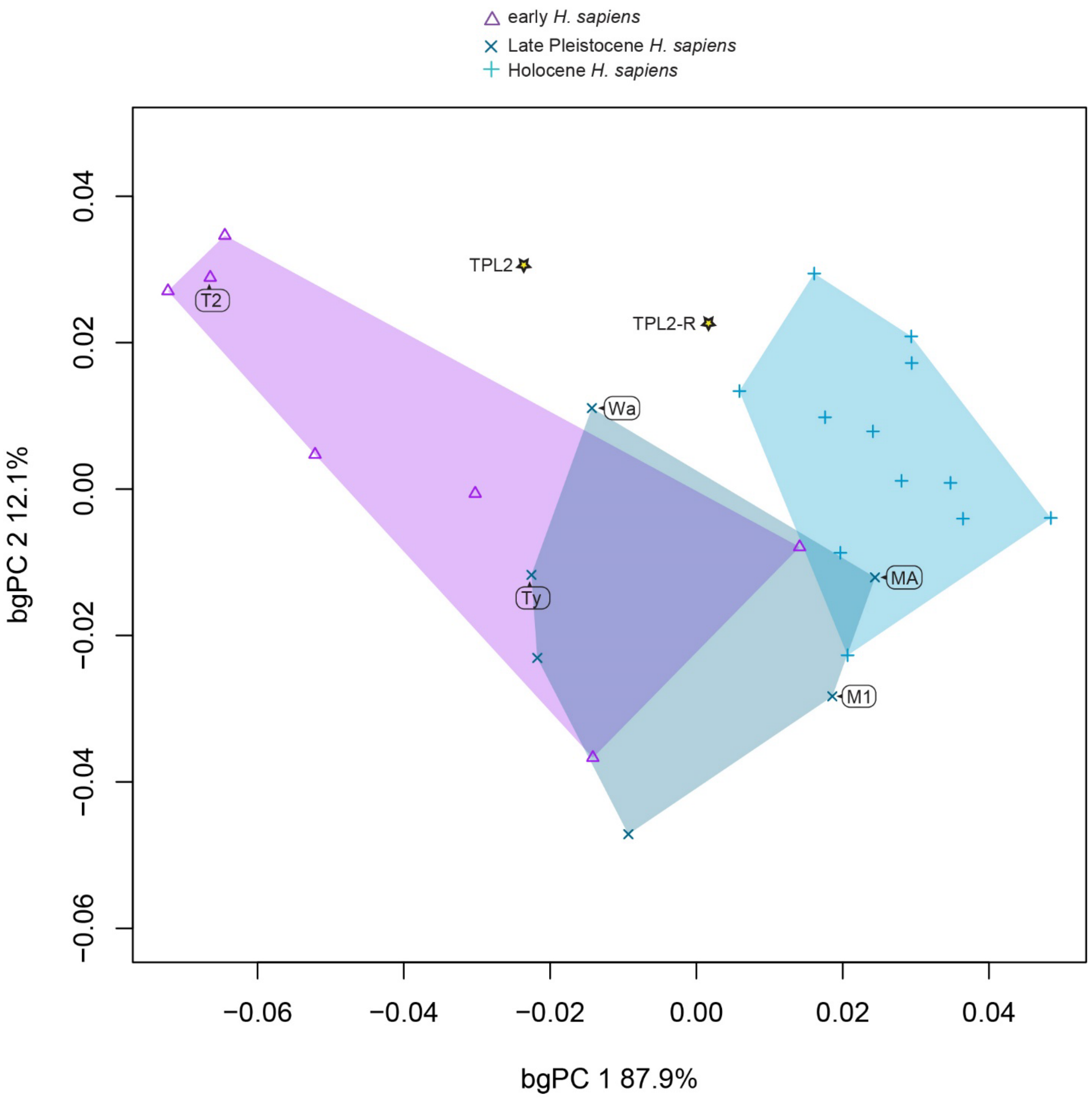

Supplementary Figure 15. Between group PCA (bgPCA) of mandibular shape in *H. sapiens*. TPL 2 reconstructions (TPL2, TPL2-R) were projected into the plot. Labels: Minatogawa A (MA) and 1 (M1); Tabun C 2 (T2); Tianyuandong (Ty); and Wadjak 2 (Wa). Source data are provided as a Source Data file.

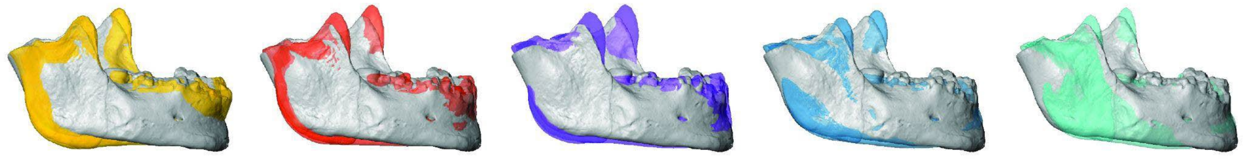

Supplementary Figure 16. TPL 2 mandible superimposed onto group mean shapes. TPL 1 (gray) superimposed on group mean shapes, anterior (A) and lateral (B) views (yellow: *H. erectus*; red: Neanderthal; purple: early *H. sapiens*; blue: Late Pleistocene Asian *H. sapiens*; green: Holocene *H. sapiens*).

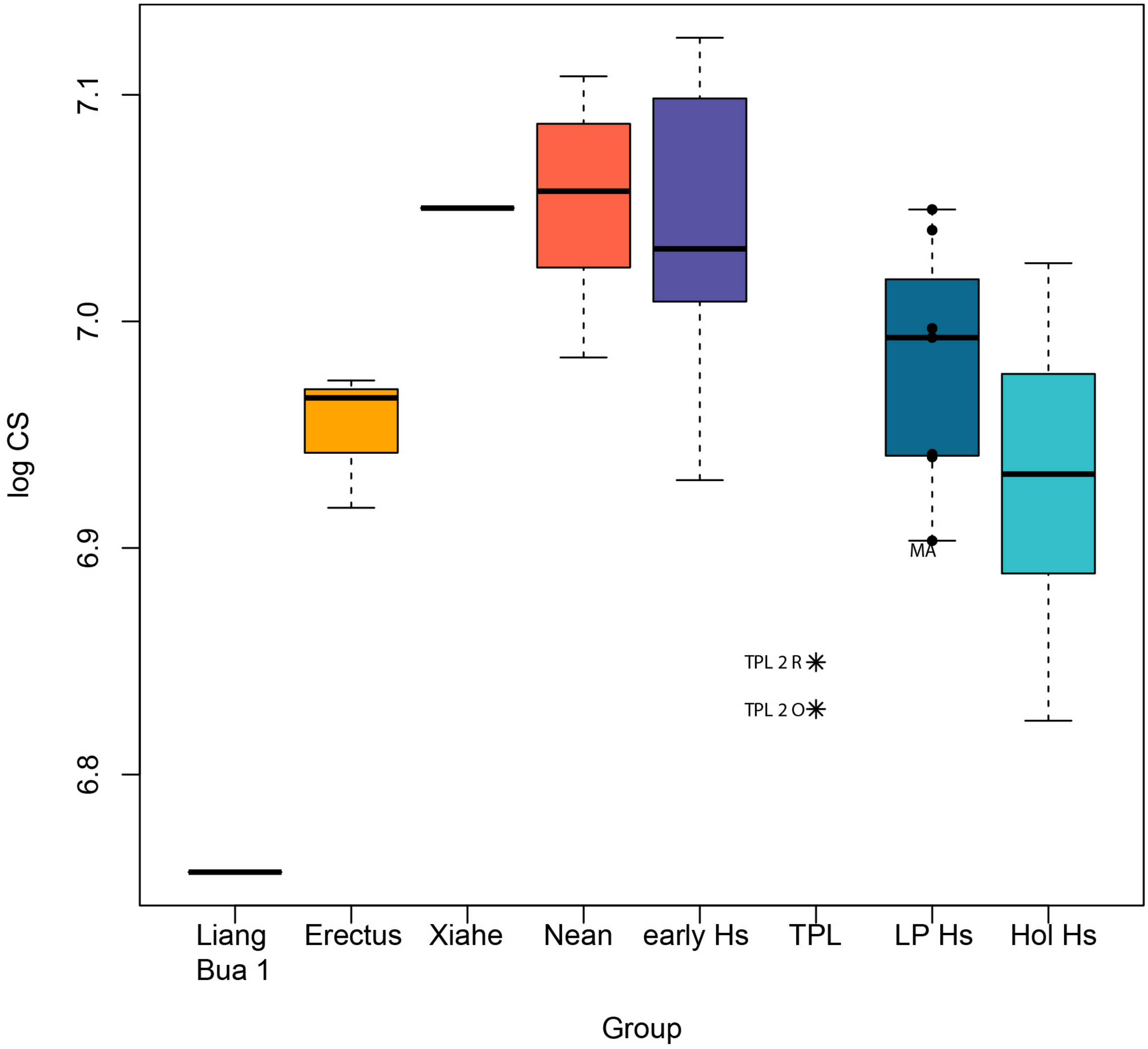

Supplementary Figure 17. TPL 2 mandible centroid sizes. Box plots depicting log centroid size for each group (*H. floresiensis* [Liang Bua 1] n = 1; *H. erectus* [Erectus] n = 3; Denisovan [Xiahe] n = 1; Neanderthal [Nean] n = 10; early *H. sapiens* [early Hs] n = 7; Tam Pà Ling hominins [TPL 2] n = 2; Late Pleistocene *H. sapiens* [LP Hs] n = 6; Holocene *H. sapiens* [Hol Hs] n = 12). Horizontal lines represent the median of each group. Boxes show the interquartile range (IQR, 25th to 75th percentile). Whiskers extend to 1.5 times IQR. Outliers are represented by circles; TPL 2 original reconstruction (TPL 2 O) and new reconstruction (TPL 2 R), Minatogawa A (MA). Source data are provided as a Source Data file.

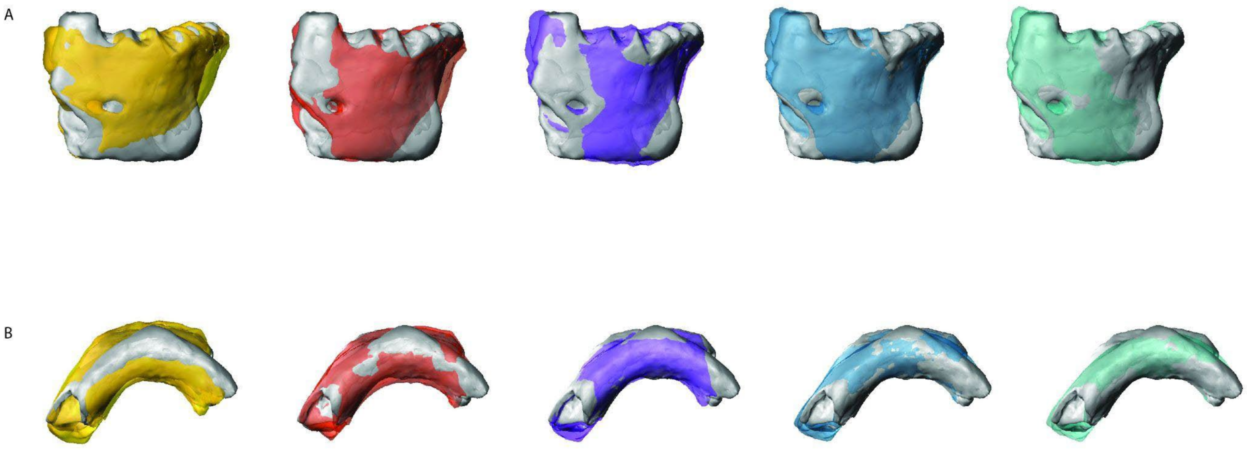

Supplementary Figure 18. TPL 3 mandible superimposed onto group mean shapes. TPL 1 (gray) superimposed on group mean shapes, anterior (A) and lateral (B) views (yellow: *H. erectus*; red: Neanderthal; purple: early *H. sapiens*; blue: Late Pleistocene Asian *H. sapiens*; green: Holocene *H. sapiens*).

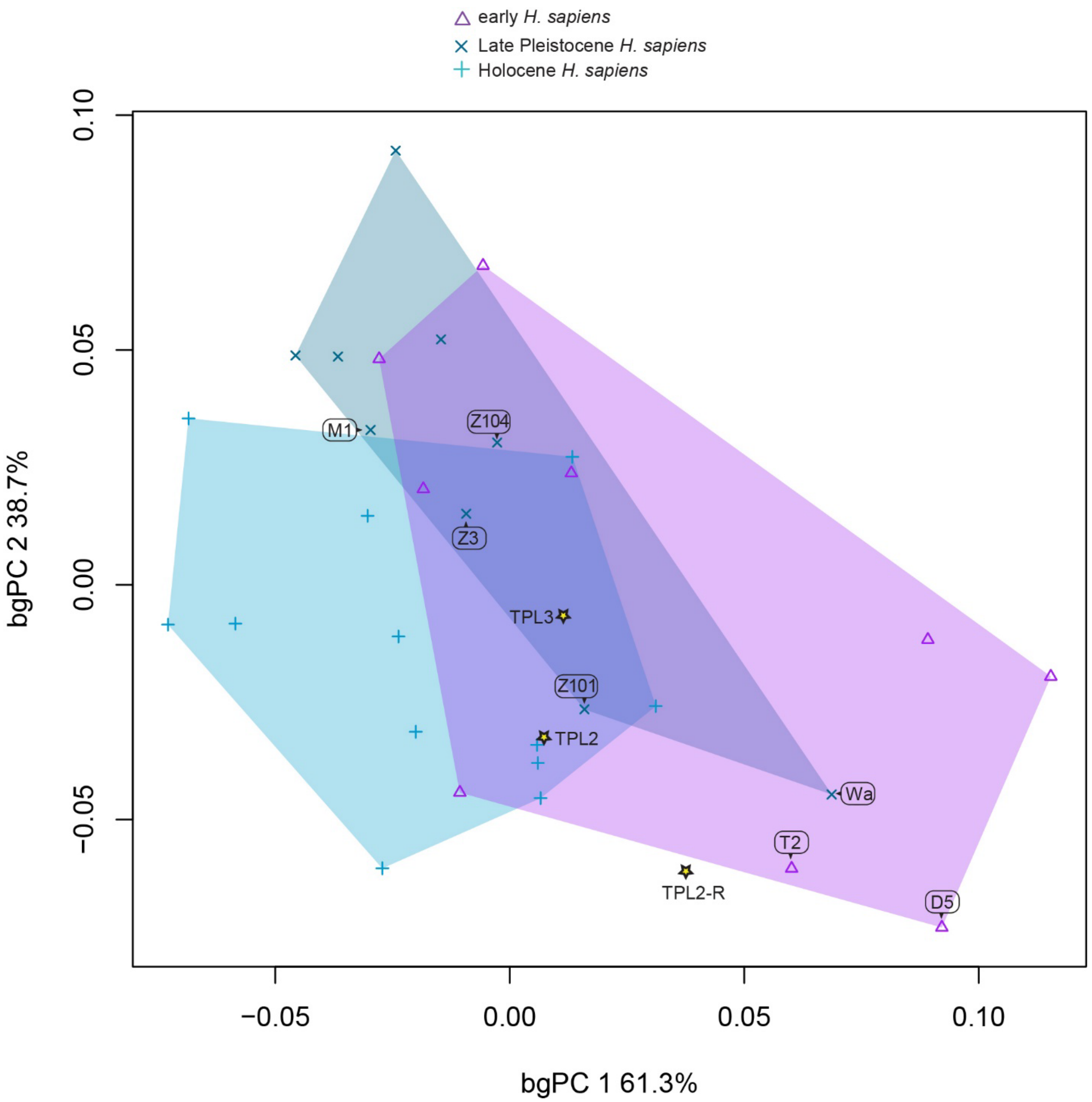

Supplementary Figure 19. Between group PCA (bgPCA) of anterior corpus shape in *H. sapiens*. TPL 2 reconstructions (TPL2, TPL2-R), TPL 3, and Zhirendong 3 were projected into the plot. Labels: Dar es-Soltane 5 (D5); Minatogawa 1 (M1); Tabun C 2 (T2); Wadjak 2 (Wa); Zhirendong 3 (Z3); Zhoukoudian Upper Cave 101 (Z101) and 104 (Z104). Source data are provided as a Source Data file.

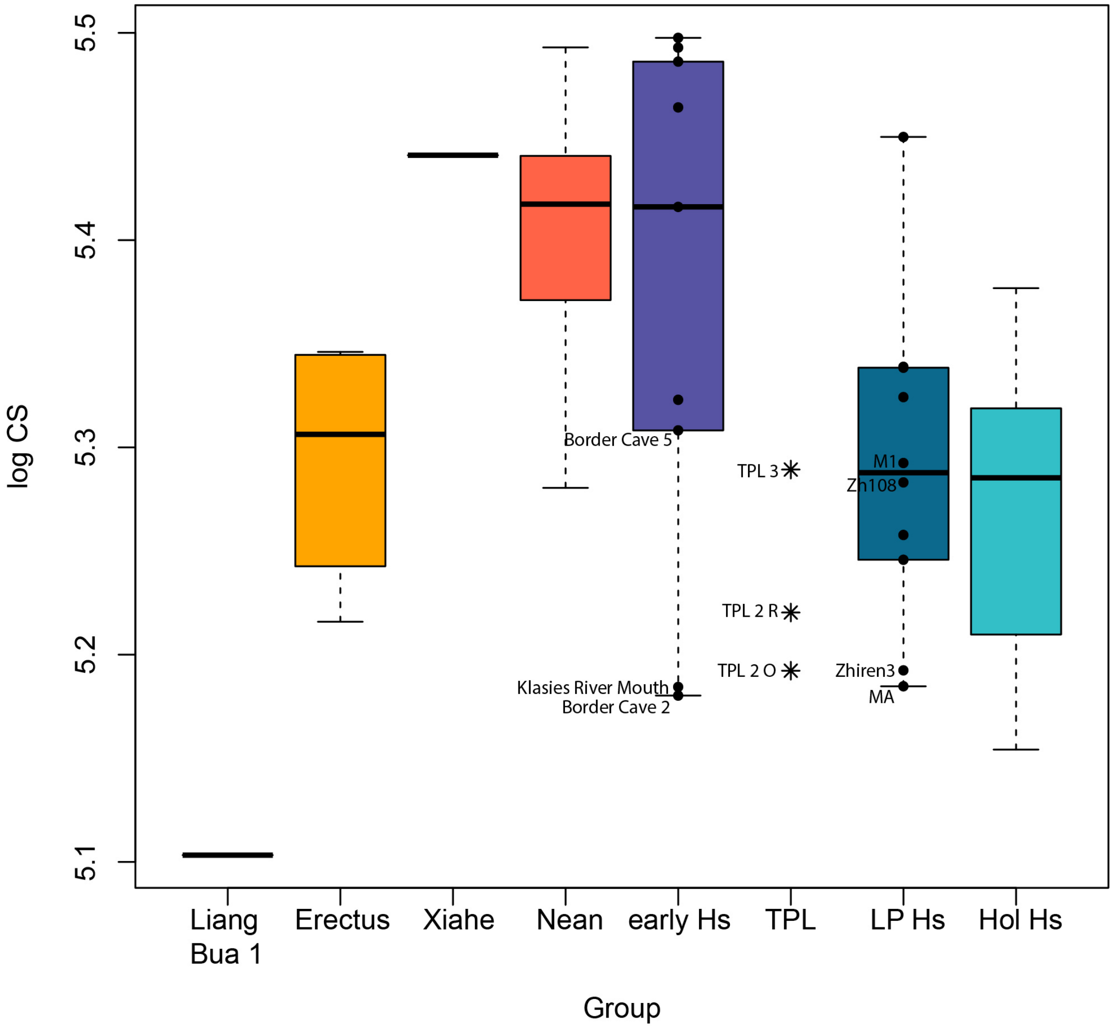

Supplementary Figure 20. TPL 3 anterior corpus centroid sizes. Box plots depicting log centroid size for each group (*H. floresiensis* [Liang Bua 1] n = 1; *H. erectus* [Erectus] n = 4; Denisovan [Xiahe] n = 1; Neanderthal [Nean] n = 15; early *H. sapiens* [early Hs] n = 9; Tam Pà Ling hominins [TPL 2 and TPL 3] n = 3; Late Pleistocene *H. sapiens* [LP Hs] n = 9; Holocene *H. sapiens* [Hol Hs] n = 12). Horizontal lines represent the median of each group. Boxes show the interquartile range (IQR, 25th to 75th percentile). Whiskers extend to 1.5 times IQR. Outliers are represented by circles; TPL 2 original reconstruction (TPL 2 O) and new reconstruction (TPL 2 R), Minatogawa A (MA), 1 (M1), and Zhoukoudian 108 (Zh 108). Source data are provided as a Source Data file.

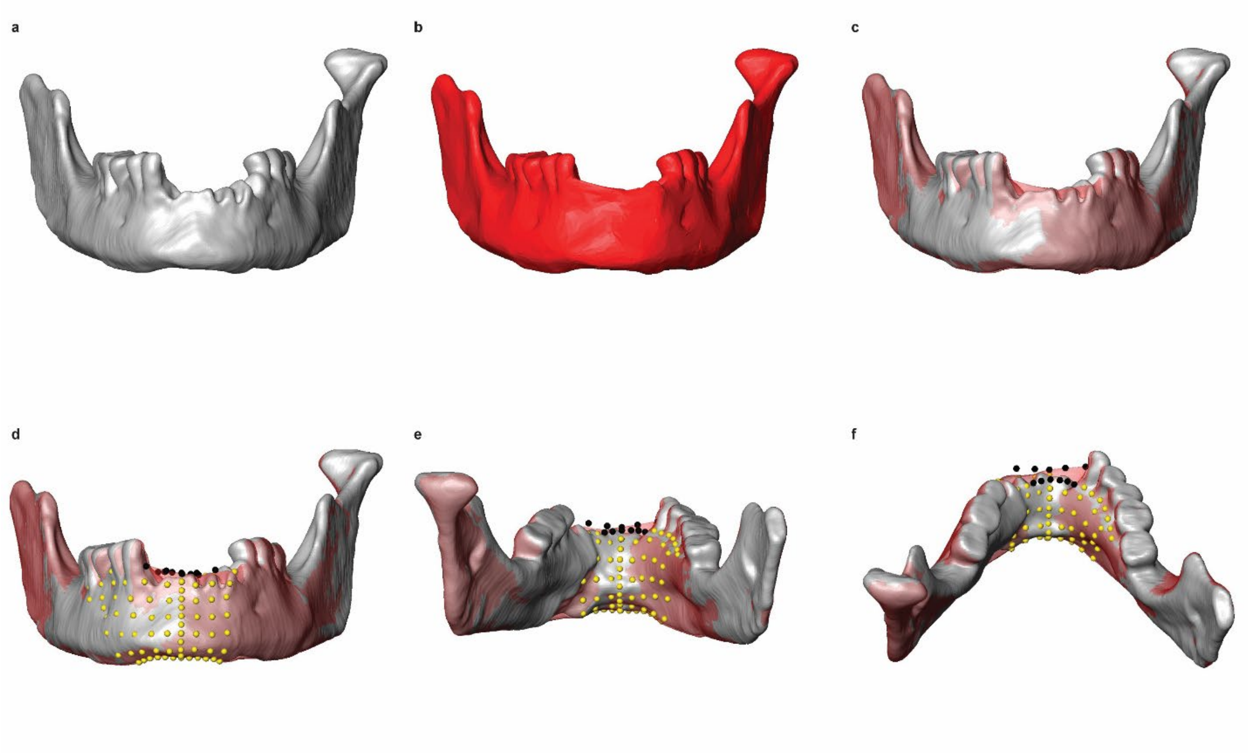

Supplementary Figure 21. Virtual reconstruction of the Minatogawa A mandible. The anterior alveolar region of Minatogawa A was virtually reconstructed and several landmarks in this region were estimated: a) the original scan with damage shown in the anterior alveolar region; b) the reconstructed scan in which the alveolar region has been reconstructed by using the hole filling algorithm in Geomagic Studio; c) the original (gray) and reconstructed versions (red) superimposed; d) anterior view of the anterior corpus landmark dataset on the original and reconstructed versions; e) posterior view of the anterior corpus landmark dataset on the original and reconstructed versions; f) superior view of the anterior corpus landmark dataset on the original and reconstructed versions. Of the 133 landmarks in the anterior corpus data set (yellow landmarks), 11 were estimated (black landmarks) using a thin plate spline (TPS) interpolation (see Methods).

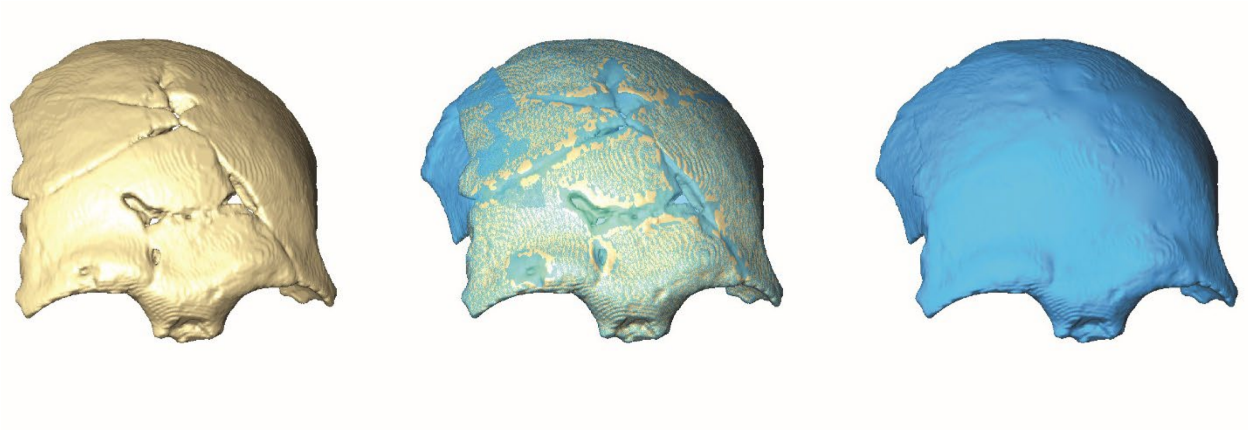

Supplementary Figure 22. TPL 1 frontal virtual reconstruction. The TPL 1 frontal bone was discovered in several pieces and manually reconstructed. It is nearly complete, missing some bone on the right side around the temporal line and small fragments near bregma and the frontal squama. We mirror-imaged the more complete left side (left) and merged it with the corresponding region on the right side (center, right). All visible cracks and holes on the bone were filled and smoothed in Geomagic Studio.

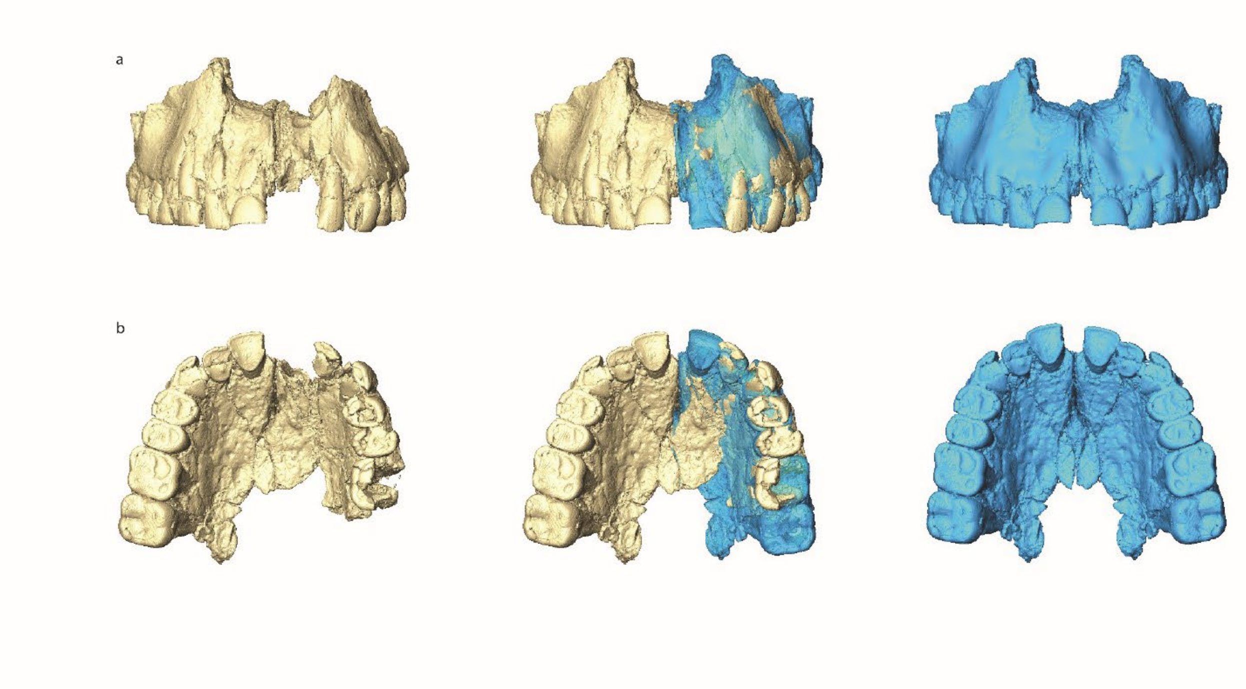

Supplementary Figure 23. TPL 1 maxilla virtual reconstruction. The TPL 1 maxilla consists of a lower left and right side (left), preserving the inferior nasal aperture, anterior nasal spine, alveolar process, left nasoalveolar clivus, palate and most of the dentition. The right side is in better condition, especially at the nasoalveolar clivus, and it preserves some of the inferior infraorbital plate including the inferior extension of the canine fossa. To achieve a more complete maxilla, the better-preserved right side was mirrored-imaged, and aligned to the left side (left center) according to the preserved morphology and the midline palatal suture.

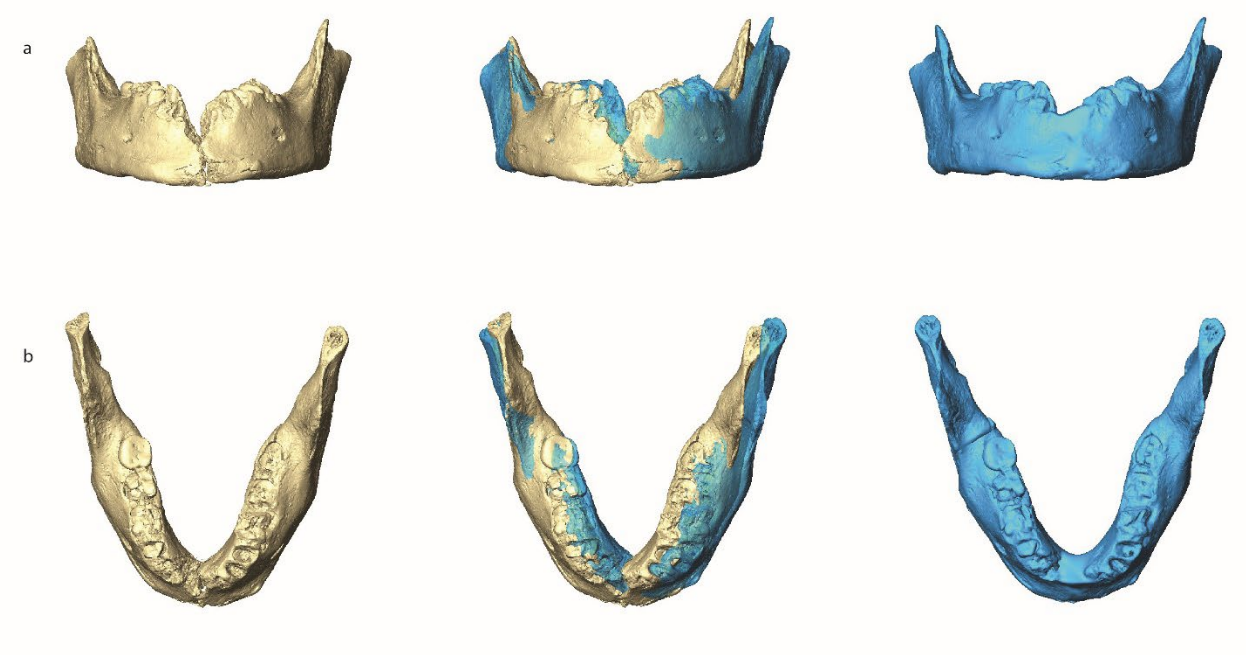

Supplementary Figure 24. TPL 2 mandible virtual reconstruction. Original reconstruction (left) and new reconstruction (right; see Methods), aligned to the original and mirror-imaged (center, right).

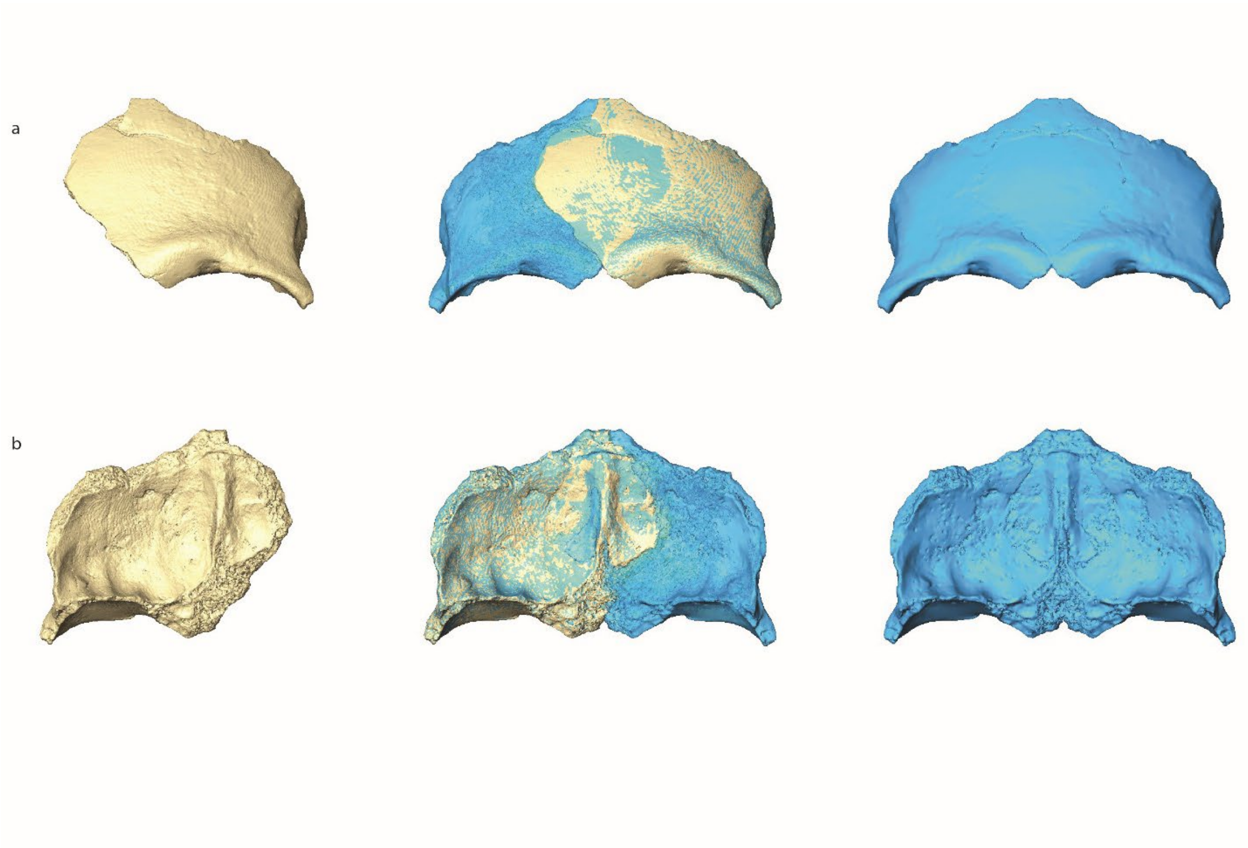

Supplementary Figure 25. TPL 6 frontal virtual reconstruction. Three different reconstructions of TPL 6 were made by reflecting its mirror image along the midsagittal plane (see Methods). Each reconstruction differs by rotating a few millimeters antero-inferiorly; (a) exterior surface, (b) interior surface.

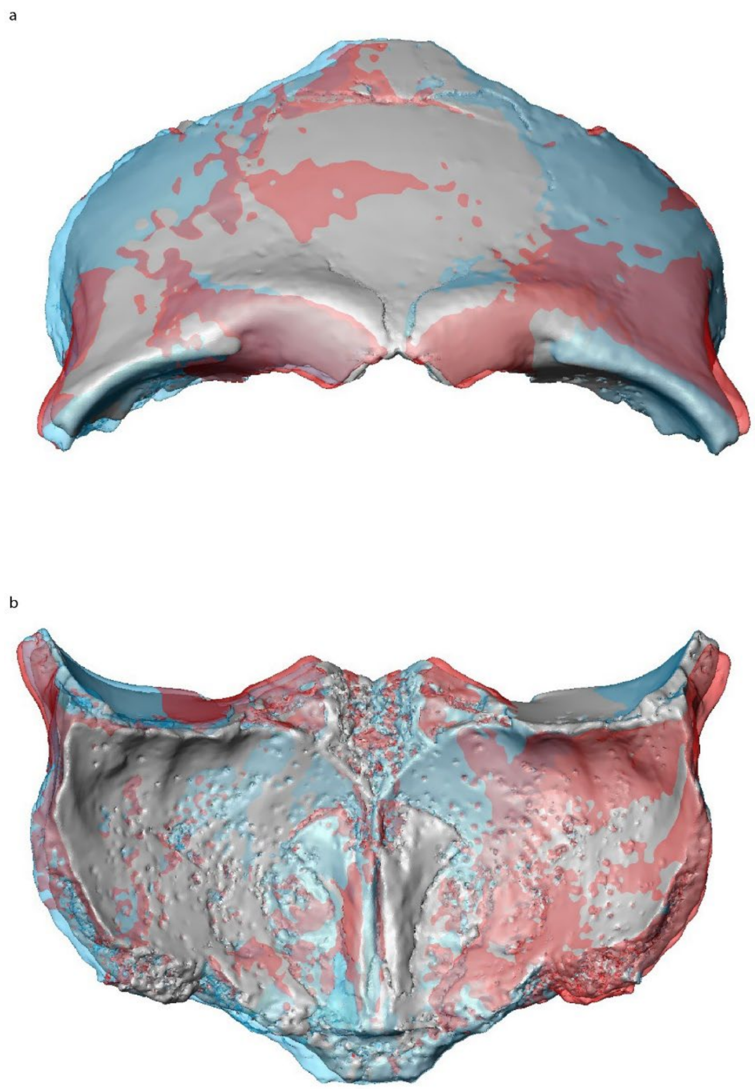

Supplementary Figure 26. TPL 6 frontal virtual reconstructions superimposed. Three different reconstructions of TPL 6 were made (see Methods): reconstruction 1 – a simple reflection mirror image along the midsagittal plane (red), and reconstruction 2 and 3 – rotating the frontal a few millimeters antero-inferiorly (blue and gray, respectively). Reconstruction 1 has the most projecting lateral brow ridge and reconstruction 2 the widest frontal bone. To keep the two halves in correct anatomical positions, all reconstructions were aligned along the frontal crest.

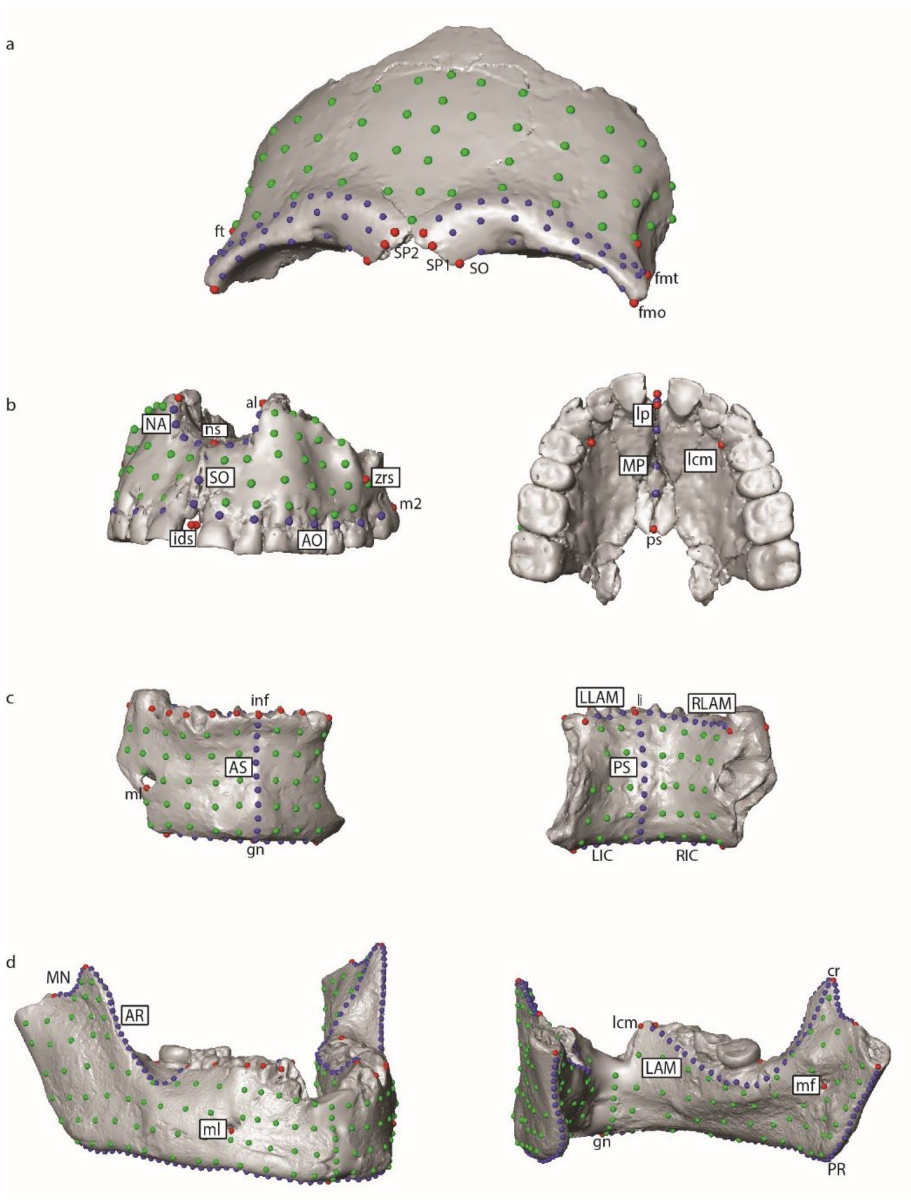

Supplementary Figure 27. Landmarks and semilandmarks used in the geometric morphometric analyses. (a) frontal; (b) maxilla; (c) anterior corpus; (d) mandible. Landmarks are red, curve semilandmarks in blue, and surface semilandmarks in green. Curves are abbreviated in capital letters and landmarks in lower case. See Supplementary Table 9 for landmark and curve and surface semilandmark definitions.

Supplementary Table 1. Results of the cross-validated linear discriminant analysis (LDA) and posterior probabilities using the *H. sapiens* sample and three groups (early, Late Pleistocene, and Holocene). The highest classification rates occurred in the mandible, followed by the maxilla, frontal, mandible, and anterior corpus. Highest probabilities in bold.

Dataset	Correct classification	Posterior probability early *H. sapiens*	Posterior probability LP *H. sapiens*	Posterior probability Hol. *H. sapiens*
**Frontal**	70.0%			
TPL 1		27.7%	**64.7%**	7.6%
TPL 6 R1		0%	34.0%	**66.0%**
TPL 6 R2		0%	18.0%	**82.0%**
TPL 6 R3		0%	18.7%	**81.3%**
**Maxilla**	70.3%			
TPL 1		**88.0%**	10.5%	1.4%
**Mandible**	72.0%			
TPL 2		2.4%	2.2%	**95.4%**
TPL 2 R		0%	0.2%	**99.8%**
**Anterior Corpus**	44.8%			
TPL 2		6.3%	30.6%	**63.1%**
TPL 2 R		27.3%	16.6%	**56.1%**
TPL 3		**39.2%**	29.3%	31.6%

1 Saurin, E. Carte géologique du Laos, feuille Luang Prabang. Publication du service geographique national du Vietnam (1961).
2 Hernandez, V. P. C., S., L., Bacon, A-M., Duringer, P., Westaway, K., Ponché, & J-L., B., S., Demeter, F., Morley, M.W. Preliminary microstratigraphic observations from Tam Pà Ling cave, Laos: Situating humans within the changing tropical environment in *Indo-Pacific Prehistory Association Congress* (Chang Mai, Thailand, 2022).
3 Ellwood, B. B. *et al.* Magnetic susceptibility applied as an age–depth–climate relative dating technique using sediments from Scladina Cave, a Late Pleistocene cave site in Belgium. *Journal of Archaeological Science* **31**, 283-293 (2004). https://doi.org:https://doi.org/10.1016/j.jas.2003.08.009
4 Lowe, K. M. *et al.* Using Soil Magnetic Properties to Determine the Onset of Pleistocene Human Settlement at Gledswood Shelter 1, Northern Australia. *Geoarchaeology* **31**, 211-228 (2016). https://doi.org:https://doi.org/10.1002/gea.21544
5 Heller, F. & Evans, M. E. Loess magnetism. *Reviews of Geophysics* **33**, 211-240 (1995). https://doi.org:https://doi.org/10.1029/95RG00579
6 Maher, B. A. Magnetic properties of modern soils and Quaternary loessic paleosols: paleoclimatic implications. *Palaeogeography, Palaeoclimatology, Palaeoecology* **137**, 25-54 (1998). https://doi.org:https://doi.org/10.1016/S0031-0182(97)00103-X
7 Dearing, J. *Environmental Magnetic Susceptibility Using the Bartington MS2 System (ed.2)*. (Chi Publishing, 1999).
8 Bacon, A.-M. *et al.* Nam Lot Southeast Asian sites revisited: Zooarchaeological and isotopic evidences. *Palaeogeography, palaeoclimatology, palaeoecology* **512**, 132 (2018). https://doi.org:10.1016/j.palaeo.2018.03.034
9 Milano, S. *et al.* Environmental conditions framing the first evidence of modern humans at Tam Pà Ling, Laos: A stable isotope record from terrestrial gastropod carbonates. *Palaeogeography, Palaeoclimatology, Palaeoecology* **511**, 352-363 (2018). https://doi.org:10.1016/j.palaeo.2018.08.020
10 Yamoah, K. A. *et al.* A muted El Niño-like condition during late MIS 3. *Quaternary Science Reviews* **254**, 106782 (2021). https://doi.org:https://doi.org/10.1016/j.quascirev.2020.106782
11 Marwick, B. & Gagan, M. K. Late Pleistocene monsoon variability in northwest Thailand: an oxygen isotope sequence from the bivalve Margaritanopsis laosensis excavated in Mae Hong Son province. *Quaternary Science Reviews* **30**, 3088-3098 (2011). https://doi.org:https://doi.org/10.1016/j.quascirev.2011.07.007
12 Dean, W. E. Determination of carbonate and organic matter in calcareous sediments and sedimentary rocks by loss on ignition; comparison with other methods. *Journal of Sedimentary Research* **44**, 242-248 (1974). https://doi.org:10.1306/74d729d2-2b21-11d7-8648000102c1865d
13 Nelson, D. W. & Sommers, L. E. in *Methods of Soil Analysis* 961-1010 (1996).
14 Heiri, O., Lotter, A. F. & Lemcke, G. Loss on ignition as a method for estimating organic and carbonate content in sediments: reproducibility and comparability of results. *Journal of Paleolimnology* **25**, 101-110 (2001). https://doi.org:10.1023/A:1008119611481
15 Demeter, F. *et al.* Anatomically modern human in Southeast Asia (Laos) by 46 ka. *Proc Natl Acad Sci U S A* **109**, 14375-14380 (2012). https://doi.org:10.1073/pnas.1208104109
16 Tappen, M. Bone Weathering in the Tropical Rain Forest. *Journal of Archaeological Science* **21**, 667-673 (1994).
17 Bacon, A.-M. *et al.* Late Pleistocene mammalian assemblages of Southeast Asia: New dating, mortality profiles and evolution of the predator–prey relationships in an environmental context. *Palaeogeography, Palaeoclimatology, Palaeoecology* **422**, 101-127 (2015). https://doi.org:https://doi.org/10.1016/j.palaeo.2015.01.011

18 Duringer, P., Bacon, A.-M., Sayavongkhamdy, T. & Nguyen, T. K. T. Karst development, breccias history, and mammalian assemblages in Southeast Asia: A brief review. *Comptes Rendus Palevol* **11**, 133-157 (2012). https://doi.org:https://doi.org/10.1016/j.crpv.2011.07.003

19 Gifford, D. P. Taphonomy and Paleoecology: A Critical Review of Archaeology's Sister Disciplines. *Advances in Archaeological Method and Theory* **4**, 365-438 (1981).

20 Lyman, R. L. *Vertebrate Taphonomy*. (Cambridge University Press, 1994).

21 Ross, A. H. & Cunningham, S. L. Time-since-death and bone weathering in a tropical environment. *Forensic Science International* **204**, 126-133 (2011). https://doi.org:https://doi.org/10.1016/j.forsciint.2010.05.018
