# Supplementary material for: Early presence of Homo sapiens in Southeast Asia by 86–68 kyr at Tam Pà Ling, Northern Laos
Source: Nat Commun. 2023 Jun 13;14:3193. doi: 10.1038/s41467-023-38715-y (PMC10264382; doi:10.1038/s41467-023-38715-y)
Supplement: Supplementary file 3 — Description of Additional Supplementary Files [file 41467_2023_38715_MOESM3_ESM.pdf]

**Descriptions of Additional Supplementary Information**

**Supplementary Data 1**. Profile 1 magnetic susceptibility (MS) and loss on ignition (LOI) data.

**Supplementary Data 2.** Profile 2 magnetic susceptibility (MS) and loss on ignition (LOI) data.

**Supplementary Data 3.** Procrustes distances (PD) between TPL 1 and 6 frontal shape and all samples in the study (CG = Cau Giat; LC = Lang Cuom; THS = Tam Hang South; THN = Tam Hang North).

**Supplementary Data 4.** Procrustes distances (PD) between TPL 1 maxilla shape and all samples in the study (CG = Cau Giat; LC = Lang Cuom; THS = Tam Hang South; THN = Tam Hang North).

**Supplementary Data 5.** Procrustes distances (PD) between TPL 2 original and reconstructed (Recon) mandible shape and all samples in the study (THS = Tam Hang South).

**Supplementary Data 6.** Procrustes distances of TPL 2 and 3 anterior corpus shape (THS = Tam Hang South).

**Supplementary Data 7.** Fossil and recent human scans used in this study; underlined specimens were casts; M = male, F = female; U = unknown.

**Supplementary Data 8.** Landmarks, curves, and surface semilandmarks used in the analyses. Midline landmarks (i.e., non-bilateral) italicized. [1]One end of curve defined with curve semilandmark and set as a true landmark when sliding.

**Supplementary Code 1. Script written for OxCal (version 4.2) used for Bayesian modeling of samples from Tam Pà Ling (https://c14.arch.ox.ac.uk/oxcal.html); script also available in a usable zipped folder in Supplementary Code 1).**
