# Supplementary material for: Early presence of Homo sapiens in Southeast Asia by 86–68 kyr at Tam Pà Ling, Northern Laos
Source: Nat Commun. 2023 Jun 13;14:3193. doi: 10.1038/s41467-023-38715-y (PMC10264382; doi:10.1038/s41467-023-38715-y)
Supplement: Supplementary file 5 — Supplementary Code 1 [file 41467_2023_38715_MOESM5_ESM.zip › Oxcal Code for TPL.docx]

Options()

{

Resolution=500;

};

Plot()

{

Sequence()

{

Boundary("base");

Phase("OSL15")

{

Date("TPOSL15 - pIR", N(calBP(80000), 10000));

Date("TPOSL15 - fines", N(calBP(86000), 12000));

Date("US teeth 1", N(calBP(71000), 1500));

Date("US-ESR teeth 1", N(calBP(84000), 8000));

};

Boundary("1");

Phase("OSL14")

{

Date("US teeth 2", N(calBP(67000),1800));

Date("TPLOSL14 pIR", N(calBP(74000),11000));

Date("TPLOSL14 fines", N(calBP(63000),12000));

};

Boundary("2");

Phase("OSL13")

{

Date("TPLOSL13 pIR", N(calBP(71000),5000));

Date("TPLOSL13 fines", N(calBP(65000),20000));

};

Boundary("3");

Phase("OSL12")

{

Date("TPLOSL12 pIR", N(calBP(67000),5000));

};

Boundary("4");

Phase("OSL10")

{

Date("TPLOSL10 pIR", N(calBP(70000),8000));

Before("TPLOSL10 OSLSG", N(calBP(48000),5000))

{

};

};

Boundary("5");

Phase("OSL3")

{

Date("TPLOSL3 pIR", N(calBP(56000),6000));

Before("TPLOSL3 OSLSG", N(calBP(48000),5000))

{

};

Before("TPLOSL3 14C", N(calBP(40000), 3000))

{

};

};

Boundary("6");

Phase("OSL2")

{

Date("TPLOSL2 pIR", N(calBP(46000), 6000));

Date("TPLOSL2 OSLSG", N(calBP(46000), 5000));

Before("TPLOSL2 US bone", N(calBP(44000), 5000))

{

};

After("TPLOSL2 US carb", N(calBP(64000), 1000))

{

};

};

Boundary("7");

Phase("OSL1")

{

Date("TPLOSL1 OSLSG", N(calBP(46000), 4000));

Date("TPLOSL1 pIR", N(calBP(43000), 7000));

After("TPLOSL1 US bone", N(calBP(64000), 6000))

{

};

Before("TPLOSL1 14C", N(calBP(40000), 3000))

{

};

};

Boundary("8");

Phase("OSL4")

{

Date("TPLOSL4 OSLSG", N(calBP(32000), 3000));

After("TPLOSL4 14C", N(calBP(39000), 1000))

{

};

};

Boundary("9");

Phase("OSL5")

{

After("TPLOSL5 14C", N(calBP(36000), 1000))

{

};

Date("TPLOSL5 OSLSG", N(calBP(22000), 2000));

};

Boundary("10");

Phase("OSL6")

{

Date("TPLOSL6 OSLSG", N(calBP(13000), 2000));

After("TPLOSL6 14C", N(calBP(25000), 1000))

{

};

};

Boundary("11");

Phase("OSL7")

{

Date("TPLOSL7 OSLSG", N(calBP(12000), 1000));

Before("TPLOSL7 14C", N(calBP(2800), 20))

{

};

};

Boundary("12");

Phase("OSL8")

{

Date("TPLOSL8 OSLSG", N(calBP(2400), 30));

Before("TPLOSL8 14C", N(calBP(1100), 20))

{

};

};

Boundary("top");

};

};
