# Supplementary material for: Early presence of Homo sapiens in Southeast Asia by 86–68 kyr at Tam Pà Ling, Northern Laos
Source: Nat Commun. 2023 Jun 13;14:3193. doi: 10.1038/s41467-023-38715-y (PMC10264382; doi:10.1038/s41467-023-38715-y)
Supplement: Supplementary file 6 — Reporting Summary [file 41467_2023_38715_MOESM6_ESM.pdf]

nature portfolio

Corresponding author(s): Fabrice Demeter and Laura Shackelford

Last updated by author(s): 05/03/2023

Reporting Summary

Nature Portfolio wishes to improve the reproducibility of the work that we publish. This form provides structure for consistency and transparency in reporting. For further information on Nature Portfolio policies, see our Editorial Policies and the Editorial Policy Checklist.

Statistics

For all statistical analyses, confirm that the following items are present in the figure legend, table legend, main text, or Methods section.

n/a	Confirmed	
☐	☒	The exact sample size (*n*) for each experimental group/condition, given as a discrete number and unit of measurement
☐	☒	A statement on whether measurements were taken from distinct samples or whether the same sample was measured repeatedly
☒	☐	The statistical test(s) used AND whether they are one- or two-sided<br>*Only common tests should be described solely by name; describe more complex techniques in the Methods section.*
☐	☒	A description of all covariates tested
☒	☐	A description of any assumptions or corrections, such as tests of normality and adjustment for multiple comparisons
☐	☒	A full description of the statistical parameters including central tendency (e.g. means) or other basic estimates (e.g. regression coefficient) AND variation (e.g. standard deviation) or associated estimates of uncertainty (e.g. confidence intervals)
☐	☒	For null hypothesis testing, the test statistic (e.g. *F*, *t*, *r*) with confidence intervals, effect sizes, degrees of freedom and *P* value noted<br>*Give P values as exact values whenever suitable.*
☐	☒	For Bayesian analysis, information on the choice of priors and Markov chain Monte Carlo settings
☒	☐	For hierarchical and complex designs, identification of the appropriate level for tests and full reporting of outcomes
☒	☐	Estimates of effect sizes (e.g. Cohen's *d*, Pearson's *r*), indicating how they were calculated

Our web collection on statistics for biologists contains articles on many of the points above.

Software and code

Policy information about availability of computer code

Data collection	Data were acquired from CT scans, surface scans (NextEngine, Minolta Vivid 910, and Breuckmann optoTOPHE) or photogrammetry (Nikon D600, Agisoft PhotoScan Professional v. 1.2.0), and landmarks were collected using Landmark Editor v.3.0.0.6
Data analysis	3D data were analyzed with Avizo 7.1, Geomagic Studio 2014 v. 3.0, Landmark Editor v. 3.0.0.6, RStudio v. 1.4.1717, using the R libraries geomorph v.4.0.2, Morpho v. 2.9

For manuscripts utilizing custom algorithms or software that are central to the research but not yet described in published literature, software must be made available to editors and reviewers. We strongly encourage code deposition in a community repository (e.g. GitHub). See the Nature Portfolio guidelines for submitting code & software for further information.

Data

Policy information about availability of data

All manuscripts must include a data availability statement. This statement should provide the following information, where applicable:

- Accession codes, unique identifiers, or web links for publicly available datasets
- A description of any restrictions on data availability
- For clinical datasets or third party data, please ensure that the statement adheres to our policy

The Tam Pà Ling fossil remains are housed at the National Museum under the responsibility of the Ministry of Information and Culture of Lao PDR.
Surface scans of the Tam Pà Ling fossils are publicly available in the Human Fossil Record archive (https://human-fossil-record.org). Source data are provided in the Supplementary Information/Source Data file.

Human research participants

Policy information about studies involving human research participants and Sex and Gender in Research.

Reporting on sex and gender	not relevant for this study
Population characteristics	not relevant for this study
Recruitment	not relevant for this study
Ethics oversight	not relevant for this study

Note that full information on the approval of the study protocol must also be provided in the manuscript.

Field-specific reporting

Please select the one below that is the best fit for your research. If you are not sure, read the appropriate sections before making your selection.

☐ Life sciences ☐ Behavioural & social sciences ☒ Ecological, evolutionary & environmental sciences

For a reference copy of the document with all sections, see nature.com/documents/nr-reporting-summary-flat.pdf

Ecological, evolutionary & environmental sciences study design

All studies must disclose on these points even when the disclosure is negative.

Study description	Morphological study of the early modern human cranial remains from Tam Pà Ling within an updated secured chronological context
Research sample	Late Pleistocene cranial human remains from Tam Pà Ling were analyzed by geometric morphometric methods and compared with large sample of Early Pleistocene to Holocene Homo specimens, including Homo erectus, Middle Pleistocene Homo, Neanderthals, Denisovans, H. floresiensis, early modern humans as well as Late Pleistocene and Holocene H. sapiens. The Late Pleistocene fossil sample was chosen because they overlap in time and geography with the Tam Pà Ling fossils, and the older Middle Pleistocene fossils and more recent Holocene H. sapiens were included to provide a larger context and to polarize the shape variation.
Sampling strategy	3D surfaces of the fossil and comparative specimens were acquired by CT scan, surface scan and photogrammetry. No sampling strategy was used, rather the sample size was determined by availability. The fossil sample, although limited, exceeds the sample sizes in many published paleoanthropological studies.
Data collection	3D landmark data was collected from surface models using the software Landmark Editor v. 3.0.0.6. All cranial data was collected by Sarah Freidline and all mandibular data was collected by Inga Bergmann.
Timing and spatial scale	The Tam Pà Ling human remains were discovered between 2010 and 2018. The specimens were scanned between 2011 and 2018, and the landmark data was collected in 2018. There was no gap in collection periods.
Data exclusions	No data was excluded
Reproducibility	The methods included in this study have been previously tested and published on numerous occasions and fully demonstrated to be reliable and replicable. This study has been replicated multiple times and all attempts at replication were successful. All source data are provided in the SI document which allows full reproducibility.
Randomization	Not relevant here as the study is based on a limited fossil sample. Allocation to the groups was based on taxonomic identity of the fossil and extant hominin specimens/samples included in the study.
Blinding	No blinding was necessary in this study. Not applicable to palaeontology/palaeoanthropology.

Did the study involve field work? ☒ Yes ☐ No

Field work, collection and transport

Field conditions	Field work activities were conducted in the frame of the international paleoanthropological mission at Tam Pà Ling, directed by F. Demeter and L. Shackelford. This is an annual fieldwork conducted before the rainfall season that comprises the excavation of the main site and the prospecting of new areas surrounding Tam Pa Ling cave. All this work is conducted in close collaboration with the Ministry of Information, Culture and Tourism of Lao PDR and also with the support and help of the villagers from Long Gua Ppa.
Location	Tam Pà Ling is located in the Huà Pan province, Laos. The tower karst in which the cave was formed is positioned on the south-eastern side of P'ou Loi Mountain at 1170 m of altitude and its GPS coordinates are 20°12′31″N 103°24′35″E.
Access & import/export	Export permits were officially granted between 2010 and 2018 to conduct all the analyses included in the study by the Ministry of Information, Culture and Tourism of Lao PDR.

Disturbance	No disturbance to report

Reporting for specific materials, systems and methods

We require information from authors about some types of materials, experimental systems and methods used in many studies. Here, indicate whether each material, system or method listed is relevant to your study. If you are not sure if a list item applies to your research, read the appropriate section before selecting a response.

Materials & experimental systems

n/a	Involved in the study
☒	☐ Antibodies
☒	☐ Eukaryotic cell lines
☐	☒ Palaeontology and archaeology
☒	☐ Animals and other organisms
☒	☐ Clinical data
☒	☐ Dual use research of concern

Methods

n/a	Involved in the study
☒	☐ ChIP-seq
☒	☐ Flow cytometry
☒	☐ MRI-based neuroimaging

Palaeontology and Archaeology

Specimen provenance	The Tam Pà Ling fossils were excavated from the eponym cave in the Huà Pan province, Laos. Permit to conduct this research had been granted by the Ministry of Information, Culture and Tourism of Lao PDR since 2010.
Specimen deposition	The Tam Pà Ling fossil remains are housed at the National Museum under the responsability of the Ministry of Information, Culture and Tourism of Lao PDR.
Dating methods	The dating methods applied toto date the sediment (OSL), charcoals (14C) and faunal teeth (U-series and ESR) are extensively described in the method section of the main text.

☒ Tick this box to confirm that the raw and calibrated dates are available in the paper or in Supplementary Information.

Ethics oversight	No ethical approval or guidance was required as this study was not dealing with living humans or animals.

Note that full information on the approval of the study protocol must also be provided in the manuscript.
